# Epiblast lumenogenesis is not a mammalian-specific trait

**DOI:** 10.1038/s41467-026-73768-9

**Published:** 2026-06-03

**Authors:** Antonia Weberling, Natalia A. Shylo, Hannah Wilson, Melainia McClain, Richard Kupronis, Alex Muensch, Suzannah A. Williams, Florian Hollfelder, Paul A. Trainor

**Affiliations:** 1 All Souls College, University of Oxford, Oxford, United Kingdom; 2 Nuffield Department of Women’s and Reproductive Health, University of Oxford, Oxford, United Kingdom; 3 Department of Biochemistry, University of Cambridge, Cambridge, United Kingdom; 4 Stowers Institute for Medical Research, Kansas City, MO USA; 5 Department of Biological and Biomedical Sciences, Rowan University, Glassboro, NJ USA; 6 Department of Cell Biology and Physiology, University of Kansas Medical Center, Kansas City, KS USA

**Keywords:** Epithelial-mesenchymal transition, Gastrulation, Evolutionary developmental biology, Embryology, Mesoderm

## Abstract

Epiblast lumenogenesis is a hallmark of mammalian embryogenesis, and crucial for the subsequent processes of anterior-posterior patterning and gastrulation. Based on avian model-organisms, reptile epiblasts are thought to form a monolayered flat disc to undergo these developmental events. Here, we report that the squamate, veiled chameleon (*Chameleo calyptratus*), exhibits epiblast lumenogenesis prior to anterior-posterior patterning. Using SEM, immunofluorescence, and histology techniques, we demonstrate that chameleon epiblast lumenogenesis occurs via a purse-string-like mechanism involving the formation and constriction of concentric rings of supracellular actin cables around the epiblast. Through expression analyses of *Nodal1*, *Nodal2*, *Cerberus*, *Lefty*, *Brachyury*, *Wnt3A*, and *Bmp2*, and immunostaining for Brachyury, we uncovered a *Wnt3A*- and Brachyury-positive ring at the edge of the epiblast concomitant with lumenogenesis. Furthermore, our data suggest that anterior-posterior patterning in veiled chameleons may occur independently of Cerberus and Lefty. These processes result in chameleon embryos exhibiting human embryo-like morphology, despite 300 million years of evolutionary separation. Collectively, we show that pre-gastrulation epiblast lumenogenesis is not mammalian-specific but has also evolved in some non-avian reptiles.

## Abstract (web summary)

Weberling et al. characterise veiled chameleon pre-oviposition development from fertilisation to the initiation of gastrulation and show that pre-gastrulation lumenogenesis occurs via a purse string mechanism resulting in human-like embryo morphology.

## Introduction

Epiblast lumen formation is a hallmark of mammalian pre-gastrulation morphogenesis and is essential for amnion formation, anterior-posterior development, and gastrulation^1^. In human embryos, the epiblast lumen forms upon implantation through charge repulsion and fluid pumping in concert with cell shape changes and dynamic specific gene activity^2,3^. The initially naïve epiblast consists of unpolarised pluripotent embryonic stem cells that subsequently establish apical-basal polarity and form a monolayered epithelium surrounding a central lumen. Simultaneously, these stem cells transition from naïve pluripotency to a primed state^4,5^. The proximal side of the epiblast underlying the trophoblast then becomes squamous and differentiates into the amnion^6,7^. Epiblast lumenogenesis has been reported to be critical for anterior-posterior patterning and initiation of gastrulation in mice with Bmp4 being secreted by the extraembryonic ectoderm into the proamniotic cavity to induce Wnt3 and Nodal expression in the epiblast^1^. This in turn defines the distal visceral endoderm (DVE), which expresses Nodal antagonists Cerberus1, Lefty1, Goosecoid (Gsc) and Hex^8^. Migration of the DVE to the epiblast-extraembryonic ectoderm border establishes the anterior-posterior axis^8^. In human embryos, CERBERUS1 and LEFTY1 were recently shown to establish an anterior hypoblast signalling centre^9^. However, the full signalling cascade remains to be uncovered as the extraembryonic trophoblast layer is not in contact with the amniotic cavity.

For decades, the chicken embryo has served as the primary organism for modelling (pre-)gastrulation human embryogenesis and is also thought to represent avian and non-avian development more generally^10^. During chicken embryogenesis, the epiblast and hypoblast form a 2-dimensional flat bilaminar disk through thinning of a multilayered blastoderm^11–13^. The anterior-posterior axis is established during this thinning process, through Nodal signalling and the expression of its antagonists such as Lefty and Cerberus^14,15^. However, the chicken embryo lacks lumenogenesis.

Here we describe pre-oviposition development in the veiled chameleon (*Chameleo calyptratus*), a scaled, non-avian reptile (squamate) species that is an emerging model for studying reptile gastrulation and the evolution of developmental mechanisms^16–18^. Unlike other squamates, chameleon embryos are pre-gastrula stage at the time of oviposition^16,18,19^, and are laid in clutches of up to 90 roughly time-matched eggs year-round^20^. The veiled chameleon genome was recently annotated^21^ and karyotyped^22^ making it a genetically tractable model organism. While its post-oviposition development gains increasing attention, chameleon pre-oviposition development was last studied in the 1930s^23,24^.

**Fig. 1:**
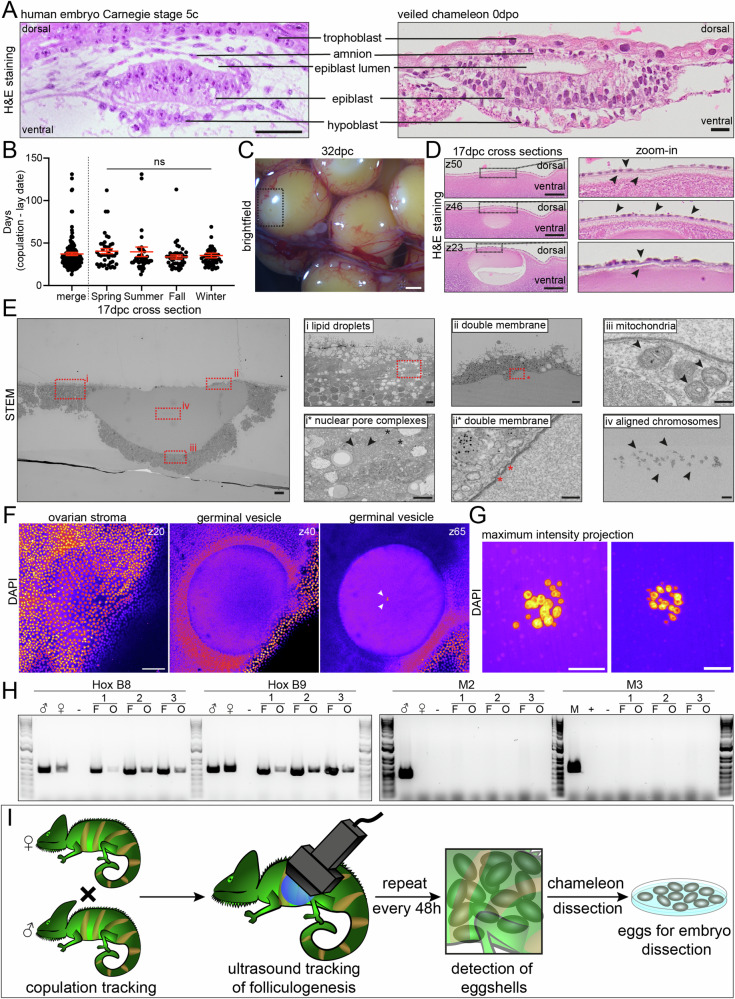
Germinal Vesicle morphology in veiled chameleons. **A** H&E staining of the cross-section of Carnegie stage 5c human embryo^7^ (left) and 0dpo veiled chameleon embryo (right). The dorsal-ventral axis of the embryos is annotated. The position of trophoblast-like, amnion, epiblast lumen, epiblast, and hypoblast are indicated. Chameleon representative of 6 embryos. Scale bar human = 50 μm. chameleon = 20 μm. **B** Timing of pre-oviposition development in the veiled chameleon. Scatter plot of overall time between mating and oviposition of 178 matings (left) and separated by season (right). Statistical analysis median ± SEM: combined: 32 ± 1.89 days, spring (41 matings): 36 ± 3.03, summer (46 matings): 29.5 ± 5.73, fall (42 matings): 32 ± 2.35, winter (49 matings): 32 ± 2.80. One-way Anova: *p*-value = 0.5063. **C** Brightfield image of mature vitellogenic follicles in the ovary at 32 dpc. Box: germinal disc with germinal vesicle. Scale bar: 2 mm. **D** H&E staining of cross sections of a germinal disc. The germinal vesicle appears as an empty sphere. Zoom in on the monolayered ovarian stroma. *n* = 3. Scale bars: 200 μm. **E** TEM of the germinal vesicle. Red squares indicate zoom-ins. Scale bar: 20 μm. Ei the yolk around the germinal vesicle is rich in lipid droplets, (Ei*) nuclear pore complexes (arrows) and Golgi apparatus (asterisks), (Eii) germinal vesicle surrounded by double membrane, (Eii*) double membrane with pores, (Eiii) mitochondria found in direct proximity to the germinal vesicle, (Eiv) aligned chromosomes in the middle of the germinal vesicles. *n* = 2. Scale bars: i/ii/iv: 2 µm, i*: 1 µm, ii*/iii: 200 nm. **F** 3D confocal imaging of DAPI-stained germinal vesicle. DNA is visible in the middle of the germinal vesicle (z65). Scale bar: 100 μm. *n* = 12. **G** Spinning Disc imaging of DNA in the centre of the germinal vesicle. The DNA appears separated into single chromosomes. *n* = 5. Scale bars 10 μm. **H** Genotyping of DNA extracted from follicles (F) and germinal vesicles (O) of 3 individual clutches (1–3). Control genes: HoxB8, HoxB9, male loci M2, M3. As controls, male DNA (♂), female DNA (♀) and a water control (−) were used. **I** Schematic of experimental set-up. Following copulation, chameleons are subjected to ultrasound every 48 h. Upon detection of specific eggshell maturity, chameleons are dissected, and the embryos are isolated and analysed. Source data of all quantitative analyses are provided as a Source Data file.

In contrast to other reptiles, we found that veiled chameleons (chameleons) form an epiblast lumen, and that chameleon embryo morphology remarkably resembles that of human embryos^7^ with a dorsal trophoblast-like layer, a ventral hypoblast layer and an epiblast surrounding a central lumen with the dorsal portion of the epiblast exhibiting squamous, amnion-like morphologies (Fig. 1A). We uncovered circular Brachyury expression and an epithelial-to-mesenchymal-like transition coinciding with lumenogenesis, prior to anterior-posterior patterning, raising the question of when gastrulation initiates. Lumenogenesis in chameleons appears to be mediated via a purse-string mechanism with supracellular actin cable constriction. Lastly, we also describe the evolutionary divergence of anterior-posterior patterning between chameleons, mammals and chickens. Taken together, our work reveals that lumenogenesis and pre-gastrulation amnion formation are not mammal-specific and may have evolved multiple times.

## Results

### Sperm storage leads to variable time between copulation and oviposition

To study chameleon pre-oviposition morphogenesis, it was necessary to first define the timing of key reproduction and early embryo developmental events. Staging conventions are commonly based on time passed since copulation, which correlates with fertilisation^1,13,25^. Chameleons lay 3–4 clutches per year^20^, and by monitoring the copulation and oviposition dates, we observed an average lay date of 37 days post copulation (dpc) (Fig. 1B). We therefore decided to isolate eggs between 17–37 dpc, but could only observe large vitellogenic follicles in the ovaries (Figs. 1C, S1A).

Each follicle contained a germinal disc with the germinal vesicle in the middle (Fig. 1C, box, S1A, boxes asterisks). Histological sections of the germinal disc show that the germinal vesicle (~ 300*μ*m diameter) (Fig. 1D) is overlaid by the ovarian stroma (Fig. 1D, arrows). We then carried out TEM of the germinal vesicle (Fig. 1E). The yolk is very rich in lipid droplets (Fig. 1Ei) and we could observe nuclear pore - and Golgi complexes (Fig. 1Ei*). The nuclear envelope exhibits nuclear pores (Fig. 1Eii, ii*) and has mitochondria in the immediate proximity (Fig. 1Eiii). Interestingly, we observed distinct structures in the middle of the germinal vesicle reminiscent of aligned chromosomes (Fig. 1Eiv). More than 20 structures could be counted, which was suggestive of a diploid set of chromosomes, since the chameleon has 24 chromosomes^21,22^. To confirm this observation, we DAPI-stained the germinal discs and found that this central structure retained the DAPI label (Figs. 1F z65, S1B z66, arrows. S1B) and was composed of over 12 DAPI-positive foci (Figs. 1G, S1C), again suggesting diploidy.

These follicles were collected at various timepoints post-mating, suggesting that fertilisation timing may not correlate with copulation. Squamates are known for short- and long-term sperm storage^26,27^, but this had not been previously described for veiled chameleons. To confirm that these follicles were indeed oocytes, we dissected the germinal vesicles of three clutches at 30–37dpc and extracted DNA of the pooled germinal vesicles (O) and immature follicles (F). Genotyping for two control genes (HoxB8/9),^28^ confirmed successful DNA extraction (Fig. 1H left). Negative genotyping for two male-specific loci (M2/3)^21^ revealed that the follicles are indeed oocytes (Fig. 1H right), confirming that chameleons store sperm.

Thus, the day of copulation cannot be used to calculate embryonic age. However, eggshell formation initiates with embryogenesis, and the density of the eggshells compared to surrounding inner organs can be detected via ultrasound and used to predict pre-oviposition stage^29^ (Figs. 1I, S1D, S2). The eggshell is initially only faintly visible (Fig. S2C) but matures into a clearly distinguishable ridge increasing in width and thickness (Fig. S2D–F). We therefore used ultrasound imaging to monitor embryo progression, enabling our investigation of pre-oviposition embryogenesis (Figs. 1I, S1D, S2).

### Cleavage divisions give rise to a multilayered embryonic shield

**Fig. 2:**
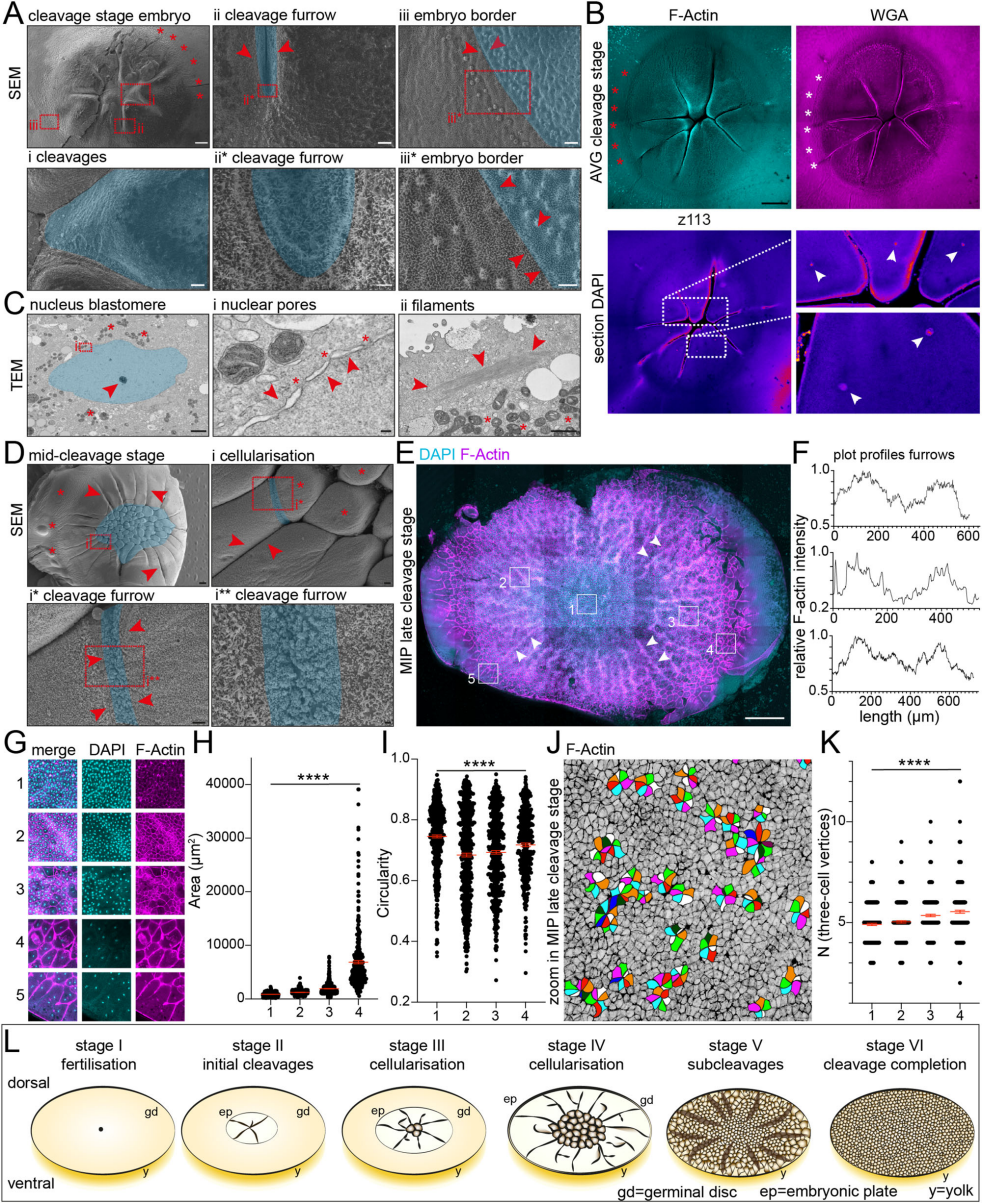
Initiation and progression of Cleavage divisions. **A** SEM early cleavage stage. embryo plate border (asterisks). (i) cleavages, initial blastomere (blue). *n* = 2. (ii/ii*) cleavage furrow highlighted in blue. (iii/iii*) embryo border, embryonic plate in blue, arrows indicate border. scale bars: cleavage stage embryo = 200 μm, i/ii/iii = 40 μm, ii* = 5 um, iii* = 20 μm. **B** Confocal microscopy, early cleavage stage. top: average intensity projection, left F-actin, right Wheat-Germ-Agglutinin. Embryo border (asterisks). bottom: DAPI staining of z-slice z113. Nascent blastomere nuclei indicated (arrows). *n* = 3. scale bar:400 μm. **C** TEM initial cleavage blastomere. Left nucleus blastomere. Nucleus (blue), nucleolus (arrow). mitochondria (asterisks). (i) Nuclear pores. Double membrane (arrows), pores (asterisks). (ii) Filaments. Filaments (arrows), mitochondria (asterisks). *n* = 2. scale bars: nucleus blastomere = 2 um, i = 100 nm, ii = 1 um. **D** SEM mid-cleavage stage. *n* = 2. Cellularised area (blue). large cleavage furrows (arrows), embryonic plate border (asterisks). (i) Cellularisation. Cellularised blastomeres (asterisks), mature cleavage furrows (arrows), immature cleavage furrow (blue). (i*/**) Cleavage furrow. Immature cleavage furrow (blue, arrows). Scale bars mid-cleavage stage 200 μm, i = 20μm, i* = 10 μm, i** = 2 um E Confocal microscopy late cleavage stage embryo. maximum intensity projection, DAPI (cyan), F-Actin (magenta). Large furrows (arrows). boxes 1–5 embryo areas. *n* = 6. scale bar:1 mm. **F** Plot profiles F-actin intensity across cleavage furrows. Each plot profile is drawn in 100 μm thickness across 2 cleavage furrows. *n* = 3. **G** zoom-ins of (**E**) DAPI (cyan) & F-Actin (magenta) staining of different areas, late cleavage stage. **H** Analysis of the surface area of embryonic cells areas 1–4. Scatter plot 3 embryos per area. Scatter plot, mean ± SEM. Statistical analysis: One-way Anova *p*-value < 0.0001, unpaired two-sided *t*-test areas 1–2, 2–3, 3–4, each *p*-value < 0.0001. **I** Quantitative Analysis of embryo cell circularity across areas 1–4. 3 embryos were analysed. Scatter plot, mean ± SEM. Statistical analysis: One-way Anova *p*-value < 0.0001, unpaired two-sided *t*-test areas 1–2 *p*-value < 0.0001, areas 2–3 *p*-value = 0.3012, areas 3–4 *p*-value = 0.0048. **J** Rosettes within the centre of the late cleavage stage. **K** quantitative analysis of three-cell vertices across areas 1–4. 3 embryos analysed. Scatter plot, mean ± SEM. Statistical analysis: One-way Anova *p*-value < 0.0001, unpaired two-sided *t*-test areas 1–2 *p*-value = 0.1323, areas 2–3 *p*-value = 0.0002, areas 3–4 *p*-value = 0.0606. **L** schematic of embryogenesis across cleavages. Source data of all quantitative analyses are provided as a Source Data file.

The initial cleavage furrows form following fertilisation in eggs covered with very thin eggshells (Figs. 2A, S3A, B). The first furrow spans the length of the embryonic plate, which can be discriminated from the germinal disc by different surface structures (Figs. 2A, S2B blue). The next cleavages initiate through furrows emanating from the embryo centre (Fig. S3Bi*) and holes at the border of the embryonic plate (Fig. S3Bii**,***). This pattern results in an initially radial cleavage pattern within the embryonic plate (Fig. 2A, Ai, i*). The embryonic plate border can be clearly observed through SEM (Fig. 2Aiii, iii*) and F-actin and WGA labelling (Fig. 2B top). Staining with DAPI revealed individual DAPI-positive foci in the individual blastomeres but no enlarged spheres as in the germinal vesicle (Fig. 2B, bottom, arrows). TEM of single blastomeres revealed the nucleus localises to the dense dorsal region (Fig. S3C, i). The staining inside the nuclear envelope exhibits characteristic density patterns suggestive of multiple nucleoli Figs. 2C, S3Ci*). The double-layered envelope contains nuclear pores (Fig. S1Ci**) and is surrounded by mitochondria and Golgi (Fig. S3Cii-ii*). Interestingly, we observed a high concentration of filaments oriented in different directions in association with cleavage furrow formation (Fig. 2Cii, S3Cii**,iii-iv).

Subsequently, the first blastomeres form while the cleavage furrows span the entire embryonic plate, which by now extends over the entire germinal disc (Figs. 2D, S3D). The cleavage divisions continue until the entire embryonic plate is composed of blastomeres (Fig. 2E). Interestingly, large radial furrows extend from the middle towards the edge of the embryonic plate even though the entire plate is already composed of blastomeres, similar to *Anolis sagrei*^30^ (Fig. 2E arrows). These furrows exhibit high actin intensity indicative of tension^31^ (Fig. 2F), and during this period, the eggshell becomes more pronounced (Fig. S3E).

Within the late cleavage stage embryo, five different areas can be distinguished. In the middle (Figs. 2E, G1, S4A), the blastomeres appear dense. In the adjacent area (2), the cells are less crowded but still homogenous. Radiating further laterally, both large and small blastomeres are found in area 3, which become even larger and more lateral in area 4. Area 5, the most lateral domain, is in direct contact with the edge of the embryonic plate and contains open cleavage furrows and multinucleated cells (Fig. 2G5, arrows). We validated these qualitative observations through quantitative analysis and observed an exponential increase in cell area and perimeter while revealing high variability in cell circularity. Central blastomeres exhibit high circularity, which decreases in blastomeres located more laterally in area 2, before increasing again in areas 3 and 4 (Figs. 2H, I, S4B–D).

During our analysis, we observed cells arranged in multicellular rosettes (Figs. 2J, S4E), a sign of increased tissue rigidity^32^. Epithelia exhibit hexagonal packaging^33^, and we observed an overall increase in three-cell vertices from areas 1–5 (Figs. 2K, S4F). This may be accounted for by the increase in size and perimeter (Figs. 2H, I, S4B–D. Taken together, we defined 6 distinct developmental stages during the initial phase of chameleon pre-oviposition embryogenesis, leading from fertilisation (stage I) to the completion of cleavage divisions with an embryo formed of small non-adherent blastomeres (stage VI) (Figs. 2L, S5).

### Epiblast lumenogenesis occurs via tissue folding

**Fig. 3:**
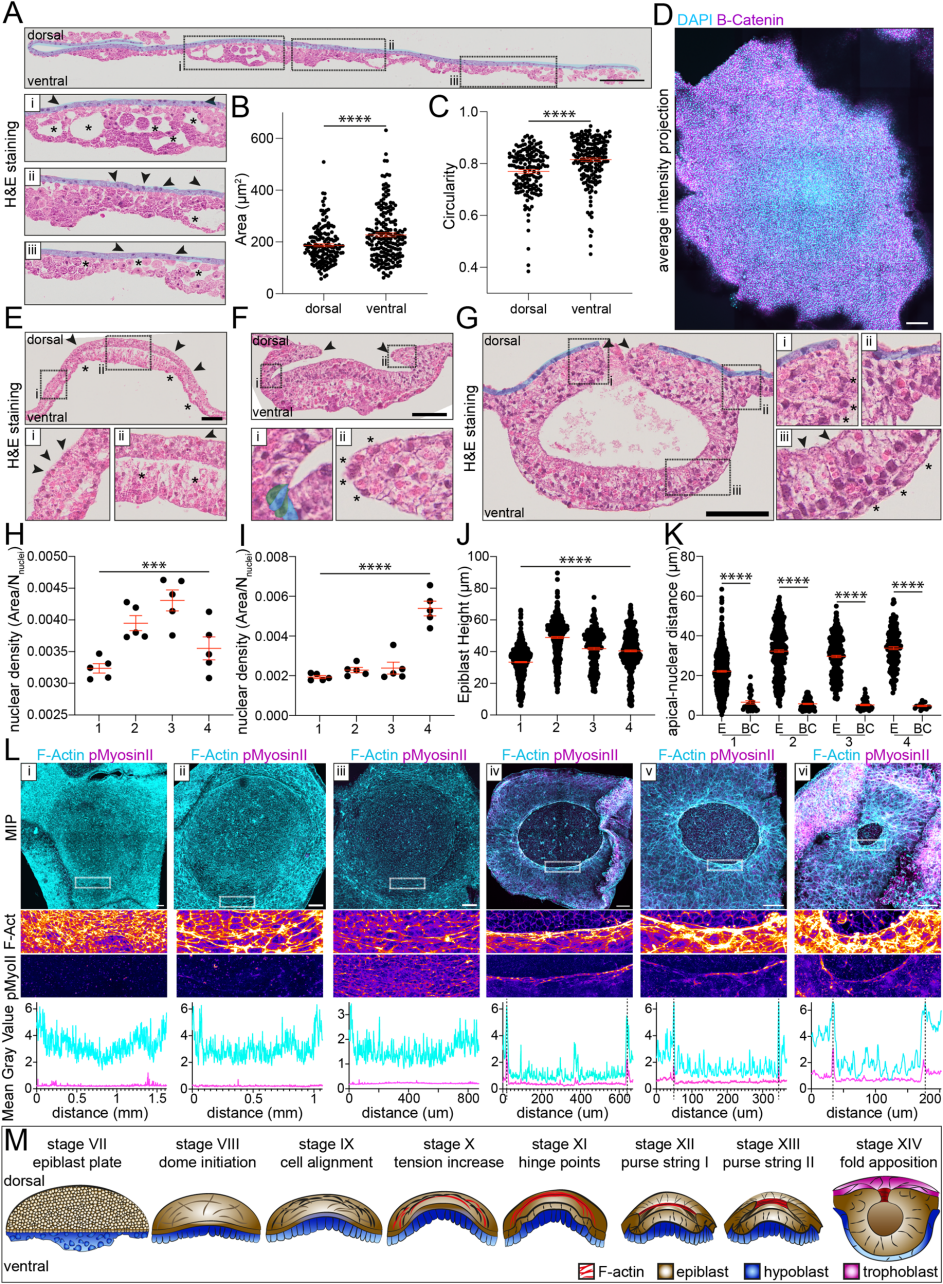
The Epiblast Lumen forms via a purse-string mechanism. **A** H&E staining of a post-cleavage stage embryo. Embryo composed of coherent dorsal tissue (squamous/cuboidal cells), the prospective epiblast (blue) and spongy ventral tissue, the prospective hypoblast. (i–iii) zoom in on different embryo regions, epiblast (blue, arrows), the hypoblast (asterisks). *n* = 3. scale bar = 200 μm. **B**, **C** Quantitative analysis of dorsal/ventral tissues for Area (**B**), Circularity (**C**), *n* = 3. Scatter plot, mean ± SEM. Statistical analysis: unpaired two-sided *t*-test. *p*-values area < 0.0001, circularity < 0.0001. **D** Confocal Imaging of post-cleavage stage embryo. Average intensity projection DAPI (cyan), β-Catenin (magenta). *n* = 3. scale bar = 200 μm. **E**–**G** H&E stainings of consecutive embryos during lumenogenesis. region of zoom-in (squares). (E/ii) epiblast (arrows), hypoblast (asterisks). (Ei) hinge-point (arrows). **F** amnion folds (arrows). (Fi) apically constricted cells in the hinge-point. (Fii) border cells (asterisks). **G** amnion fold apposition (arrows), trophoblast-like layer (blue). (Gi) apposing fold cells (asterisks). (Giii) hypoblast (asterisks), epiblast (arrows). n total = 4. All scale bars 100μm. **H**, **I** Quantitative analysis of nuclear density (tissue area/number of nuclei within tissue) of epiblast (**H**), hypoblast (**I**) during 4 lumenogenesis stages. Scatter plot, mean ± SEM. Epiblast: Statistical Analysis one-way Anova *p* = 0.0004, unpaired two-sided *t*-tests *p*-values (1–2) = 0.001, (2–3) = 0.1156, (3–4) = 0.0148. Hypoblast: Statistical Analysis one-way Anova *p* < 0.0001, unpaired two-sided *t*-tests *p*-values (1–2) = 0.0483, (2–3) = 0.7716, (3–4) = 0.0002. 4 embryos, 5 cross-sections/embryo. **J** Epiblast tissue height during 4 lumenogenesis stages. Scatter plot, mean ± SEM. Statistical analysis: one-way Anova *p* < 0.0001, unpaired two-sided *t*-tests *p*-value (1–2) < 0.0001, (2–3) < 0.0001, (3–4) = 0.1527. 4 embryos, 5 cross-sections/embryo. **K** apical-nuclear distance in epiblast (**E**) versus tip cells in lumenogenesis folds (BC). Scatter plot, mean ± SEM. Statistical analysis, unpaired two-sided t-tests of E vs BC, all *p*-values < 0.0001. 4 embryos, 5 cross-sections/embryo. **L** top row maximum intensity projections of F-actin (cyan) and phosphorylated Myosin II (pMyosinII, magenta) during lumenogenesis (i-vi) in whole embryos. Dorsal view. Squares: zoom-in regions of rows 2 (F-actin), 3 (pMyosinII). Columns iv-vi supracellular actomyosin cable (arrows). Bottom row. Plot profiles of actin (cyan) and pMyosinII (magenta) mean grey value across the length of the embryo. actomyosin cable (black lines). Fire staining (yellow-white: high signal intensity, purple-black low signal intensity). *n* = 10 consecutive embryos. Scale bars: 100 μm **M** schematic of embryogenesis during lumen formation comprising stages VII-XIV. Source data of all quantitative analyses are provided as a Source Data file.

Following the completion of cleavage divisions, the blastomeres become adhesive and form a coherent sheet^30^ (Figs. 3A, S6A). Tightly packed cells build the dorsal side of the embryo (Fig. 3Ai-iii, arrows), while the ventral side exhibits larger, loosely packed, rounded cells (Figs. 3A, S6A asterisks, 3B, C, S6B, C). This ventral population thickens in the middle of the embryo (Figs. 3A, S6A) and is more apparent through immunofluorescent imaging which illustrates the crowding of the nuclei towards the middle of the embryonic shield (Fig. 3D). The dorsal cells exhibit comparable surface areas throughout the embryo, thus, the higher density of nuclei in the middle must be associated with the ventral cell layers (Figs. 3B, S6D). These cell populations give rise to the epiblast (dorsal) and the hypoblast (ventral).

Subsequently, the embryo which was initially a flat tissue, hollows and forms a dome-like structure (Fig. 3E) similar to the brown anole^30^. Two shoulders of tissue on each edge of the epiblast (Fig. 3Ei, arrows), appear thicker than the remaining epiblast (Fig. 3Eii). Then, the side shoulders fold dorsally over the dome-shaped epiblast (Fig. 3F arrows). This tissue folding coincides with apical constriction in the hinge points cells (Fig. 3Fi). The cells at the tips of the annealing folds orient towards the tissue surface (Fig. 3Fii, asterisks), and while the folds anneal further but are not yet closed (Fig. 3G arrows), the epiblast curvature inverts from concave to convex (Fig. 3E–G). A squamous cell layer is then observed dorsally (Figs. 3G, i, ii highlight, S6E, i, ii arrows) while the remaining fold cells appear disorganised. The embryo is embedded in a multilayered tissue originally located at the outer area of the embryonic shield (Fig. 3Gii). Simultaneously, the hypoblast rearranges from a thick, spongy tissue into a thin cell layer surrounding the convex epiblast (Figs. 3Eii, F, Giii asterisks).

Measuring nuclear density in the epiblast and hypoblast at successive stages of lumenogenesis reveals an increase in epiblast nuclear density during folding, which diminishes upon fold annealing (Fig. 3H). The hypoblast nuclear density remains constant and only increases dramatically upon inversion of epiblast curvature (Fig. 3I). Epiblast height is higher in the middle versus the sides and increased slightly during folding but then decreases upon fold annealing (Fig. 3J). The apical-nuclear distance of epiblast cells is highly variable, which is indicative of a pseudostratified epithelium, while the tip cells exhibit much shorter distances to the apical surface (Fig. 3K).

We next investigated lumenogenesis through whole-mount F-actin and phosphorylated myosin II (pMyoII) staining (Fig. 3L, S7A). Upon the initiation of hollowing, the central epiblast cells were significantly smaller than the outer cells and of higher circularity (Figs. 3Li, S7Ai, S7B-J). This size difference remained throughout lumenogenesis, while the overall circularity decreased. Once the epiblast dome matured, the border cells aligned in concentric rings around the epiblast centre (Figs. 3Lii, S7Aii). Then, the border cells elongated and exhibited slightly higher actin levels while pMyoII remained low (Fig. 3Liii, S7Aiii). Next, supracellular actin rings appeared around the epiblast dome in subsequent stages (Fig. S7Aiii, iv, red lines plot profiles). The ring-cells stretched so thin that individual cells could not be distinguished (Fig. S7Aiv, v). Subsequently, these ring-cells disappeared, and instead, the epiblast was overlaid and surrounded by an actin ring in direct contact with the large outer cells (Fig. 3Lv, zoom-in).

This actin ring appears to be a supracellular actomyosin cable (Fig. 3Liv–vi). Actin and pMyoII peaked at the border between epiblast and outer cells on top (Fig. 3Liv–vi, plot profiles black lines, Fig. S7A, plot profiles red lines). This actomyosin ring constricted further over the epiblast, exhibiting even higher actin intensity than the underlying epiblast (Figs. 3Lvi, S7Avi). Taken together, we defined another 8 consecutive stages of lumenogenesis stages from the coherent embryo (stage VII) to the closely annealed, although not-yet closed epiblast lumen (stage XIV) (Figs. 3M, S5).

### Epiblast lumen closure is followed by symmetry breaking and gastrulation

**Fig. 4:**
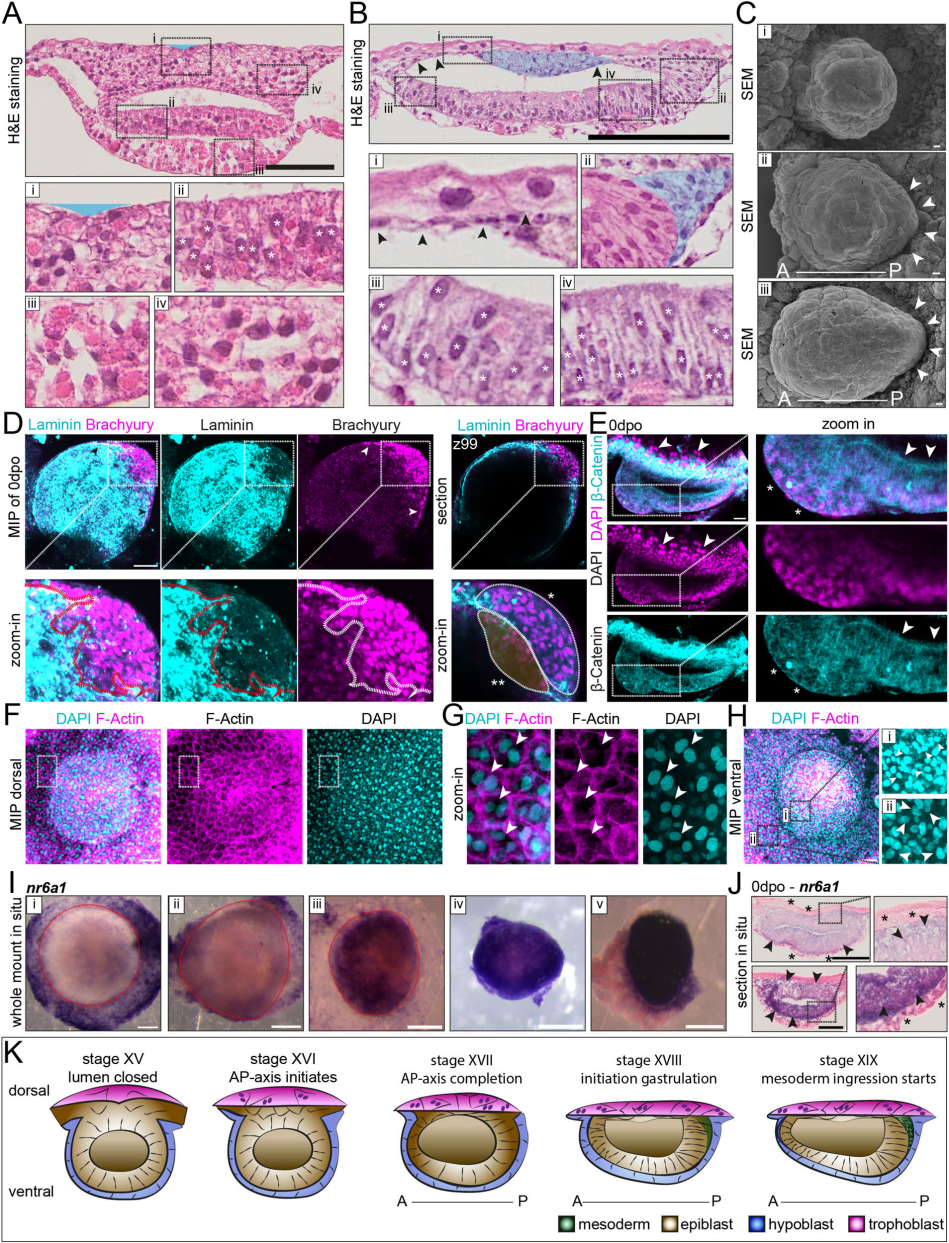
Lumenogenesis is followed by anterior-posterior patterning and gastrulation. **A** H&E staining preoviposition embryo following lumen closure. The amniotic fold fusion point visible as dorsal indentation (blue highlight, i). zoom ins: (i) amniotic fold fusion (ii) zoom in epiblast, asterisks indicate nuclei (iii) hypoblast (iv) epiblast-derived dorsal tissue shows no polarity. *n* = 2. scale bar = 200 μm. **B** H&E staining 0dpo embryo. amnion fusion remnant highlighted (blue), squamous amnion cells (arrows). zoom-ins: (i) dorsal enlarged trophoblast-like cells. ventral squamous amnion (arrows). (ii) posterior embryo and mesoderm ingression. Embryo (magenta), ingressing mesoderm (blue). (iii/iv) anterior (iii) and posterior (iv) epiblast nuclei (white asterisks). *n* = 6. scale bar = 200 μm. **C** SEM of symmetry breaking. Imaging from the ventral side. Posterior (arrows). 3 consecutive embryos. scale bars: i/iii = 20 μm, ii = 30 μm. **D** 0dpo embryo, confocal microscopy IF staining of Laminin (cyan), Brachyury (magenta). left 3 columns maximum intensity projection, zoom-ins: breach of basement membrane (dashed line). Right column single z-section. Zoom-in: *-marked outline ingressed mesoderm cells, **-marked outline mesoderm cells not yet ingressed through the basement membrane. *n* = 5. scale bar = 100 μm. **E** 0dpo embryo, confocal microscopy IF staining DAPI (magenta) β-Catenin (cyan). Double-nucleated trophoblast-like cells (arrows). zoom ins: pseudostratified epithelium in epiblast (arrows), rounded mesoderm cells following ingression (asterisks). *n* = 3. scale bar = 50 μm. **F** maximum intensity projection of dorsal side following lumen closure, DAPI (cyan), F-actin (magenta). squares indicate zoom-ins of (**G**), *n* = 5. scale bar = 100 μm. **G** zoom-ins of the trophoblast-like cell layer (**F**). Double-nucleated cells (arrows). **H** maximum intensity projection of the ventral side of the embryo following lumen closure. DAPI (cyan), F-actin (magenta) squares indicates zoom ins (i/ii). i. Zoom in on hypoblast/epiblast nuclei; arrows indicate single nuclei. ii. zoom-in on trophoblast-like nuclei. Single nuclei (arrows). *n* = 5. Scale bar = 100 μm. **I** whole mount in situ hybridisations of Nr6a1 from initiation of lumenogenesis to oviposition. Epiblast (red line). *n* = 30 consecutive embryos. All scale bars = 200 μm. **J** cross-section in situ hybridisation Nr6a1 of 0dpo embryo. dorsal top. Epiblast (arrows), extraembryonic tissues (asterisks). *n* = 2. Scale bars: top 200 μm, bottom 100 μm. **K** A schematic of embryogenesis from lumen formation to the initiation of gastrulation, comprising stages XV–XIX.

The epiblast lumen closes via the purse-string with the closure point remaining visible (Fig. 4A, Ai). The epiblast cells are columnar (Fig. 4Aii, asterisks) and overlie the hypoblast, a loosely packed pseudostratified epithelium (Fig. 4Aiii). The cell layer in between the epiblast and the emerging trophoblast-like tissue appears disorganised (Fig. 4Aiv). Upon oviposition, the trophoblast-like tissue transforms into enlarged, squamous cells overlaying highly squamous amnion-like cells (Fig. 4B, Bi, arrows) with the closed lumen still visible as a morphological tissue thickening (Fig. 4B, blue). Gastrulation is visible by spindle-shapes cells that delaminate from the epiblast and migrate between the epiblast and hypoblast (Fig. 4Bii, blue). The epiblast itself undergoes posterior thickening through apicobasal elongation of the epithelium (Fig. 4Biii, iv, asterisks). Subsequent embryo symmetry breaking and the initiation of gastrulation are evident through SEM (Fig. 4Ci–iii).

To investigate the initiation of gastrulation in more detail, we stained 0dpo embryos for Laminin and the gastrulation marker Brachyury (Fig. 4D). Laminin forms a thin basement membrane on the basal side of the epiblast, and a distinct gap at the site of gastrulation was observed (Fig. 4D, zoom ins). Brachyury-positive cells ingress from the epiblast and spread over the laminin-labelled basement membrane (Fig. 4D arrows). Two populations of Brachyury-positive cells were observed, one migrating out of the epiblast (Figs. 4D z99*, S8A) and the second within the epiblast (Figs. 4D z99**, S8A). Gastrulation involves the delamination of cells from the epiblast epithelium, and we therefore analysed 0dpo embryos for β-Catenin, to demarcate shape, and DAPI to stain nuclei, and observed the distinct morphologies of a highly columnar epiblast, and the smaller, rounded gastrulating cell population (Fig. 4E, F zoom in). Interestingly, the trophoblast-like cell population appears double-nucleated with enlarged nuclei compared to the epiblast/hypoblast populations (Fig. 4F–H arrows).

Syncytium-formation and enlarged nuclei are hallmarks of trophoblast maturation in mammals^34–36^, which this trophoblast-like population exhibits as well (Fig. 4G, H, arrows). Since no trophoblast tissue marker was specific, double nucleation and increased nucleus size can serve as morphological markers for this trophoblast-like population. We were also not successful in identifying an epiblast protein marker. Instead, we performed in situ hybridisation for *Nr6a1*, which has been reported to influence the repression of pluripotency^37–39^. *Nr6a1* was initially expressed in the cells surrounding the doming epiblast, possibly indicating a loss or decrease in pluripotency in these cells (Fig. 4I, J left). Upon completion of lumen formation, *Nr6a1* expression was downregulated in trophoblast-like tissue and instead upregulated in the epiblast (Fig. 4J, right). Histological sections confirmed that *Nr6a1* expression is restricted to the epiblast tissue, and not expressed in hypoblast and trophoblast-like cells (Fig. 4I, J). Taken together, we define 5 stages (XV-XIX) between lumen closure and initiation of gastrulation (Figs. 4K, S5).

### Circular *Brachyury* and *Wnt3a* expression occur prior to anterior-posterior patterning

After developing a morphology-based staging system for functional studies in the veiled chameleon, we investigated the signalling pathway dynamics governing anterior-posterior patterning and the initiation of gastrulation. To this end, we carried out in situ hybridisation to define the spatiotemporal expression of the known key regulators *Cerberus*, *Lefty*, *Nodal1*, *Nodal2*, *Bmp2*, *Wnt3a*, and *Brachyury* from stage VIII- XIX.

**Fig. 5:**
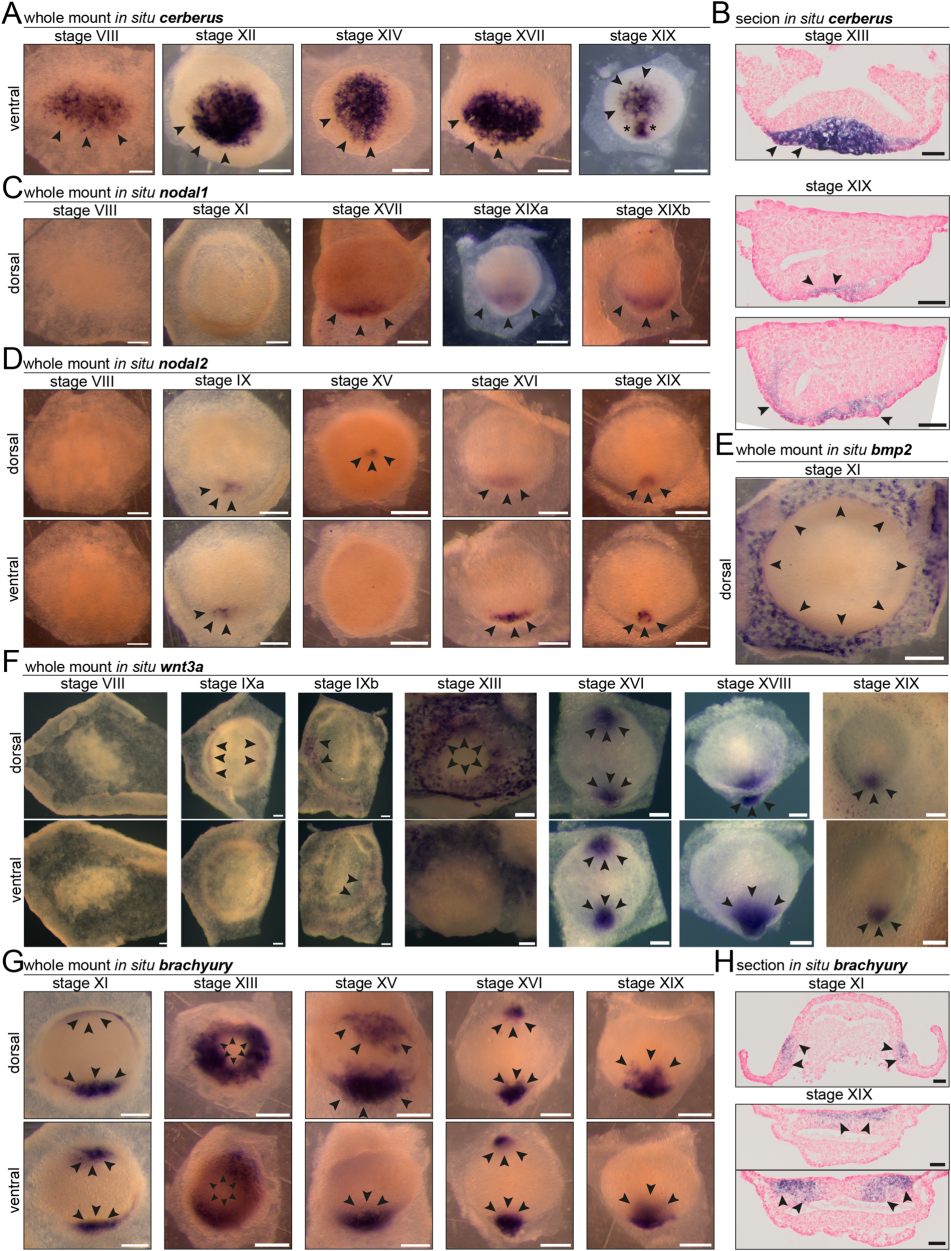
Expression patterns of anterior-posterior marker genes and gastrulation marker Brachyury. **A**–**H** gene expression analysis over time (stages VIII-XIX) through in situ hybridisations of (A/B) Cerberus, whole mount *n* = 32 consecutive embryos, cross section *n* = 2 consecutive embryos. **C** Nodal1, 30 consecutive embryos. **D** Nodal2, *n* = 33 consecutive embryos. **E** Bmp2, *n* = 27 consecutive embryos. **F** Wnt3a, *n* = 18 consecutive embryos (G/H), Brachyury, whole mount *n* = 32 consecutive embryos, cross sections *n* = 3 consecutive embryos. Arrows indicate sites of expression. asterisks (A, stage XIX) indicate the site of gastrulation. Dorsal/ventral views are indicated in the figure. **A**, **C**–**G** whole mount in situ. (B/H) cross sections. Scale bars whole mount in-situs = 200 μm, scale bars sections = 100 μm.

*Cerberus* is expressed as early as stage VIII in a broad domain in the middle of the immature hypoblast (Figs. 5A VIII–XIX, S9A VIII–XIX), similar to human embryos^9^ and remains expressed throughout lumenogenesis. Upon initiation of gastrulation, *Cerberus* expression divides into two domains, with one remaining in the middle of the embryo (Fig. 5A XIX arrows), while the other forms a distinct domain overlying the site of gastrulation (Fig. 5A XIX asterisks). Histological sections confirmed that *Cerberus* is expressed in the hypoblast (Fig. 5B, arrows) as is characteristic for this gene^8^. However, *Cerberus* is typically a marker of the anterior of the embryo, but here it is expressed in the posterior. Thus, *Cerberus* may not function as a primary regulator of anterior fate or identity in the veiled chameleon. Lefty, another anterior marker, was not detected throughout the entire time course (Fig. S9B VIII–XIX). Distinct *Lefty* expression during left-right patterning^17^ confirmed that the gene is active in chameleon embryos but not expressed during anterior-posterior patterning (Fig. S9Bi).

Focussing on the posterior markers *Nodal1* and *Nodal2*, we observed *Nodal1* expression following lumen formation in a domain demarcating the future posterior region of the embryo, which then spreads along the sides of the embryo upon gastraultion (Figs. 5C, arrows, S9C). *Nodal2* expression initiates at stage IX and localises to one domain on the side of the epiblast dome (Fig. 5D). Following lumen formation, *Nodal2* is expressed dorsally in the middle of the embryo, consistent with radial symmetry of the embryo (Fig. 5D XV). Subsequently, *Nodal2* is expressed in a posterior domain and remains in a small circular domain at the site of gastrulation (Fig. 5D stages XVI, XIX).

We then examined *Bmp2* expression, which demarcates the trophoblast-like tissue from stage VIII until completion of lumenogenesis (Figs. 5E, S9D,E). The posterior marker *Wnt3a* and gastrulation marker *Brachyury* exhibit a striking pattern (Figs. 5F, G, S9F). *Wnt3a* expression initiates at stage IX in two faint, opposing domains at the edges of the doming epiblast, a pattern replicated by *Brachyury* at stage XI (Figs. 5F, G IX, XI arrows). Upon lumenogenesis, both genes form a dorsal ring over the underlying epiblast (Fig. 5F XIII, G XIII) that gives rise to the dorsal plate of gene expression (Fig. S9F XV). Following lumen closure, *Wnt3a* and *Brachyury* are expressed in two opposing domains (Fig. 5F XVI, G XVI). After downregulation in one domain, *Wnt3a* and *Brachyury* localise to the posterior of the embryo and the site of gastrulation initiation (Fig. 5F XVIII, XIX, G XIX). Cross sections show that *Brachyury* is expressed in the epiblast-derived folds that mediate lumenogenesis (Fig. 5H, arrows). Taken together, we characterised the novel gene expression dynamics of key anterior-posterior axis and gastrulation regulators that deviate from classic mammalian and avian expression patterns.

### Circular Brachyury protein expression precedes basement membrane maturation

Our findings raise the question of when gastrulation is initiated in the veiled chameleon. Peter^23^ described mesoderm formation prior to lumenogenesis; however, it is commonly accepted that chameleon embryos are at pre-gastrula or very early gastrula stages upon oviposition^16,18^. One hallmark of gastrulation is localised basement membrane breakdown enabling ingression of the nascent mesoderm^40–42^.

**Fig. 6:**
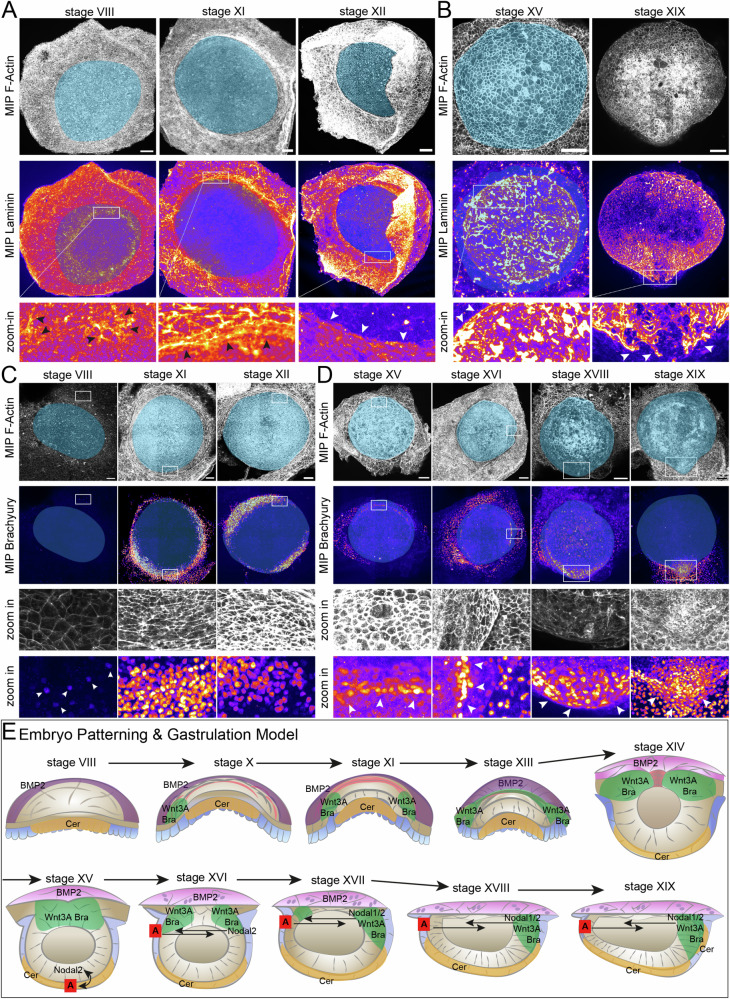
Circular Brachyury protein expression prior to basement membrane maturation. **A**, **B** Maximum intensity projection of (**A**) dorsal view of embryos stages VII-XII. **B** ventral view of embryo stages XIII-XIV. Top row F-actin. bottom row Laminin fire staining illustrating signal intensity (yellow-white: high signal intensity, purple-black: low signal intensity). Blue highlight indicates epiblast. squares highlight zoom-ins in the bottom row. Arrows indicate areas of high laminin intensity (**A**) or the site of basement membrane breach (**B**). n(A) = 7 consecutive embryos, n(B) = 6 consecutive embryos. scale bar embryo1 = 200 μm, all other scale bars 100 μm. **C**, **D** Maximum intensity projection of (**A**) dorsal view of embryos stages VIII-XII, (**B**) ventral view of embryos stages XV-XIX. Top row F-actin, Row 2 Brachyury. Blue highlights indicate the epiblast. Squares indicate zoom-in regions of the bottom 2 rows. Arrows highlight Brachyury-positive cells. n(C) = 12 consecutive embryos, n(D) = 15 consecutive embryos. All scale bars = 100 μm. **E** Final model of lumenogenesis, anterior-posterior patterning and gastrulation.

Following Laminin localisation over time, we initially observed a ring of Laminin strands oriented radially to the centre of the hollowing epiblast (Fig. 6A VIII). Laminin then forms thick filaments at the edge of the epiblast dome extending into the trophoblast-like population but not within the epiblast (Fig. 6A XI). Upon purse-string initiation, Laminin levels increase in the trophoblast-like population with low levels found in the epiblast (Fig. 6A XII). Following lumen closure, Laminin forms a thin basement membrane around the entire, radially symmetric epiblast (Fig. 6B XV), which is breached at the site of gastrulation (Fig. 6B XIX). Thus, basement membrane maturation follows lumenogenesis, and basement membrane breakdown occurs around oviposition.

Our in situ hybridisation analyses revealed gene expression patterns but not functional protein localisation. Therefore, *Brachyury* expression may occur as early as stage VIII, but the functional protein may only be present following anterior-posterior patterning. Analysing Brachyury protein expression over time, we observed low levels of Brachyury in scattered cells at the epiblast border upon initiation of epiblast hollowing (Fig. 6C VIII). Then, Brachyury protein becomes localised along half of the epiblast (Fig. S10A), developing into a ring with high levels of protein expression on two opposing sides of the embryo (Fig. 6C XI-XII). The Brachyury positive cell population changes morphology from being hexagonal to highly stretched along the amnion folds (Fig. 6C zoom-in). Upon lumen closure, Brachyury protein-expressing cells cover the entire dorsal side of the embryo and extend under the trophectoderm (Fig. 6D, S10A XV–XVIII). We also observed small domains of high Brachyury protein expression located at the edges of the epiblast. At the onset of gastrulation, Brachyury protein expression is detected only in the posterior of the embryo (Fig. 6D XVIII-XIX). Taken together, a circular domain of Brachyury protein expression prior to basement membrane maturation and anterior -posterior patterning, coincides with tissue folding during lumenogenesis (Fig. 6E).

## Discussion

Here, we describe veiled chameleon pre-oviposition morphogenesis from fertilisation to gastrulation and provide evidence of the dynamic spatiotemporal expression of key regulatory genes governing anterior-posterior patterning and gastrulation. We report several conserved features and vast divergence in epiblast morphology, anterior-posterior patterning and gastrulation. Our staging system provides a framework for future functional studies, enabling comparative analyses throughout development, in the same manner as the mouse embryo staging convention of counting embryonic days (E) ensures every reader knows what stage was analysed. This will enable the effects of in vitro culture, exposure to growth factors or inhibitors and gene deletion via CRISPR/Cas gene editing or overexpression transgenesis on morphogenesis to be determined.

The initial stages of veiled chameleon embryogenesis are highly conserved between avian and non-avian reptiles^12,30,43,44^. The apparent presence of nucleoli in the initial blastomeres may suggest active transcription^45^ raising the question of when zygotic genome activation occurs, which happens in chicken at EGK stages I-IV^46^. During cleavage divisions, the chameleon embryo is not cohesive. However, a high number of multicellular rosettes in the center of the embryo at late cleavage stages are suggestive of epithelialisation^32^. Once the embryo has become coherent (stage VII), the epiblast and hypoblast can be distinguished morphologically, and it will be interesting to investigate how compaction and lineage segregation are mediated during embryogenesis. From stage VII onwards, the chameleon embryo diverges from chicken^11–13^ and other squamates such as brown anoles^30^, asp vipers^44^, and common lizards^43^.

The epiblast undergoes lumenogenesis via a tissue folding and a purse-string mechanism to give rise to a structure morphologically very similar to the amniotic cavity in a human embryo^1,6^. While we hypothesise that the lumen closes via active constriction due to actomyosin localisation, live imaging and functional experiments such as laser ablation are required to provide proof of active constriction. Nonetheless, chameleon embryos exhibit a novel mode of amniotic cavity formation as opposed to mammalian epiblast lumens, which are formed through charge repulsion and hollowing^2,47^. Following lumenogenesis in chameleon embryos, the dorsal-most cell layer originating from outer cells of the embryonic plate exhibits enlarged nuclei and binucleation, a hallmark for trophoblast maturation^34–36^. Thus, the epiblast of early chameleon embryos may have the capacity to give rise to trophoblast-like tissue. While these cells do not form a placenta, they constitute a conserved progenitor population. Further studies will be required to understand where this trophoblast-like tissue originates and how binucleation occurs.

Human and chameleon embryo morphology appears conserved following lumenogenesis (Fig. 1A), however, we report stark divergences regarding signalling dynamics and embryo patterning. Putting all our analysis together (Fig. 6E), we propose an as yet to be identified signal antagonising *Nodal1/2* and *Wnt3a* establishes the anterior-posterior axis in the absence of anterior *Cerberus* expression and a lack of *Lefty* expression. Gastrulation has been suggested to occur prior to lumenogenesis^23^ or following lumenogenesis^16,18,19^. While we report Brachyury protein expression at earlier timepoints, we did not observe evidence for anterior-posterior patterning, which is required for successful gastrulation in amniotes in vivo, prior to lumenogenesis.

These signalling pathway dynamics underlying anterior-posterior patterning and the commencement of gastrulation are distinct from mammals, suggesting divergent evolution of the anterior-posterior patterning gene regulatory network. To our knowledge, we are the first to report pre-gastrulation, circular expression of Brachyury in amniotes. It will therefore be interesting in the future to understand whether the epiblast border cells undergo a form of epithelial-to-mesenchymal transition to enable lumen closure. Thus, future studies of cell behaviour and transcriptomics will be required. Additionally, a comparison of signalling dynamics between species with and without epiblast lumenogenesis is needed to shed light on the role of the lumen in subsequent development.

Although not reported in a study focused on placenta formation in *Mabuya mabouya*, a viviparous skink species, it is evident in sections included in that study that epiblast lumenogenesis occurs in *Mabuya mabouya* embryos^48^. Epiblast lumenogenesis, therefore, evolved in animals other than mammals and chameleons and may be a more widespread phenomenon. Considering the evolutionary distance between the veiled chameleon and *Mabuya mabouya* is 225MYA^49^, this leads us to hypothesise that epiblast lumenogenesis and pre-gastrulation amniogenesis may have evolved multiple times, similar to viviparity^50^.

## Methods

### Limitations of working with a non-model organism

Many tools that are standard for model organisms, such as mouse, chicken or frog, have not yet been established in chameleons. For example, no commercially available chameleon-specific antibodies exist. We tested a number of tissue marker antibodies, such as Oct4, Sox2, Cdx2, Gata6, and Gata4, that showed either no signal or expression patterns that diverge from what has been published for the species they have been raised against. Only for highly conserved genes such as Laminin and $\beta$-catenin could we detect signals that appear specific and were in accordance with protein localisation patterns in other species. Furthermore, we did perform gene expression analyses where we could for *Nodal1*, *Nodal2*, *Cerberus*, *Lefty*, *Brachyury*, *Wnt3A*, and *Bmp2*. In addition, common tools that enable functional experiments, such as transgenesis or chemical agonist/antagonist treatment of in vitro cultured pre-oviposition embryos, have not yet been established for chameleons and such, we cannot currently provide mechanistic proof for hypothesised cell and tissue movements.

### Chameleon husbandry

Veiled chameleons originally obtained through the pet trade were housed in accordance with SIMR IACUC protocols 2022-147 Lizards and Snakes (Squamata) and 2023-160 Squamate Development following previously published husbandry protocols^20,51^. The Stowers Institute for Medical Research is an AAALAC accredited institution^20,52^. Partially screened enclosures measuring approximately 2’ x 2’ x 4’ are used for housing and are cleaned daily. Mercury vapour and T5 lighting provide access to UV sources as well as basking opportunities. Each enclosure contains a variety of artificial and natural vines, branches and foliage. Animals are fed once daily (a variety of insects and vegetables are provided on a rotating basis) while dietary supplementation is provided every other feeding. Misting and whole-room humidification occur several times daily. Adult chameleons are housed individually except during documented mating events. Breeding-age females are provided with lay tubs containing a sand/soil/peat moss mixture. These tubs are misted as needed to maintain appropriate moisture levels. Eggs are removed for artificial incubation within 24 h of oviposition. Chameleon female ages range from 6 months to 4 years. Stud ages are between 1–4 years.

### Ultrasound

Ultrasound was performed as a non-invasive method to track folliculogenesis and egg maturation using the Visual Sonics Vevo 1100 ultrasound system with an MS550D probe. The chameleons were carefully manually restrained, and then conductive gel was placed on the lower abdomen, after which ultrasound images were taken. A minimum of 3 images was taken per side. Eggshells could be distinguished by their lighter colour as shown in Figure S2. This procedure was carried out every 48 h to monitor eggshell maturation.

### Chameleon euthanasia

Female chameleons were anaesthetised using isoflurane until the toe pinch reflex was absent. For euthanasia 50% (v/v) of a tricaine methane sulphonate (MS-222) solution was injected into the heart (1 ml per 250–300 g body weight). After the loss of coloration and apparent death, we performed secondary euthanasia in the form of heart removal (pneuomothorax). This method of euthanasia is consistent with American Veterinary Association (AVMA) guidelines.

### Chameleon dissection

The skin was opened along the midline and pinned to the sides. Then the abdominal cavity was opened, and the two uterine horns containing eggs were isolated, and each was moved to PBS. The ovaries were dissected and fixed in 4%PFA. In case ovulation had not yet taken place, the ovaries containing vitellogenic follicles were isolated and moved to PBS. All dissections were carried out in PBS.

### Embryo dissection

In cleavage-stage embryos, the eggshell has not yet matured, and the embryonic shield can be distinguished through the shell as a white oval on the yolk. The eggshell was carefully cut in half and removed from the yolk, which at this stage is still quite solid. The yolk was then turned until the embryonic shield was facing upwards. Incisions were made around the embryonic shield, and then the shield, plus an underlying layer of yolk, was isolated from the remaining yolk using needles. This yolk plate is required to maintain the integrity of the embryo, as the blastomeres are not cohesive and will otherwise separate. Post-cleavage but pre-lumenogenesis embryos are embedded in the omphalopleure, which has grown around the yolk. The volume of the yolk is slightly lower than the volume of the eggshells at these stages, which enables the removal of the eggshell halves without damaging the yolk. The embryo can be distinguished from the remaining yolk as a beige-coloured circle or oval. The yolk is carefully positioned until the embryo is facing upwards, then the omphalopleure surrounding the embryo is cut using scissors, and the embryo is peeled off with forceps. The embryo is in direct contact with the yolk, in contrast to chicken embryos.

For mature pre-oviposition embryos, the omphalopleure is attached to the eggshells. This became apparent when removing the two eggshell halves from the yolk, which had a rougher, less clean surface. The eggshells were held with one pair of forceps while a second pair of forceps was moved carefully along the eggshell to separate the omphalopleure from the eggshell. Once a portion of the omphalopleure was separated, the membrane was pulled off the eggshells using forceps. The membrane was stretched out, and the embryo was located as a solid oval or circle that was surrounded by a ring of denser cells. The embryo was cut out of the omphalopleure using needles and placed in fixative.

For 0dpo embryos, the eggs were cleaned and then cut along the long axis into two halves. As the eggs are under pressure, it is important to be careful when opening the shell. After the yolk was removed, the shells were placed into PBS. From here onwards, the procedure for mature pre-oviposition embryos was followed.

All embryos were placed in ice-cold 4% (v/v) PFA in DEPC-treated PBS and fixed overnight at 4 °C. A set of embryos from each clutch was fixed in PFA and used for immunofluorescence staining, while the rest was washed 3x in PBS on the following day, dehydrated in MeOH (25/50/75/100/100%(v/v)) and stored at −20 °C to preserve RNAs for subsequent analyses.

### DNA extraction of germinal vesicles

Germinal vesicles were dissected from the embryonic plates of oocytes and collected in one tube in PBS. A second tube was filled with some follicular tissue as a control. Following dissection, the vesicles were spun down in a table-top centrifuge, and the maximum amount of supernatant was removed. Then, 50ul of solution A (0.025 M NaOH, 0.2 mM EDTA) is added to the germinal vesicles (100ul to follicles), which were added and incubated for 50 min at 95 °C. The tubes were cooled and spun down, and then 50 ul of solution B (0.04 M Tris pH5) were added to the germinal vesicle solution (100 ul to follicles) and mixed. The DNA was stored at −20 °C. The follicles were diluted 1:5 in 10 mM Tris pH 8, and the germinal vesicles were kept as is.

### Genotyping

For genotyping, we used previously published M2 and M3 primer pairs, which target two male loci^53^. 50% of fertilised zygotes were expected to be male. No oocytes had male-specific markers. We used the following two sets of primers for autosomally located Hox genes (HoxB8 and HoxB9) as positive controls.

HoxB8F5’- CGGCTTTGTACTTGGAGAAGA

HoxB8R5’- GTGGAGTACGTGGAAACCAATA

HoxB9F5’- GGGATACCCACCAAACTCTATC

HoxB9 R5’- AAATCCAGAGACCGCCATATC

The samples were subjected to PCR together with male and female control DNA as well as one empty control ((10 ul 2 x PhireMastermix, 1 ul Primer F (10 μm), 1ul Primer R (10 μm), 1ul DNA, 7ul H_2_O) run as follows 5 min 98 °C, 35 x (98 °C 5 s, 64.8 °C 5 s, 72 °C 30 s), 72 °C 1 min. The PCR products were run on a 1% agarose gel in TAE buffer and developed on a Chemismart.

### Immunofluorescence staining

Embryos were placed on a rocker for all wash and incubation steps. Embryos were washed 3x in PBS and then permeabilised for 20 min in permeabilisation buffer (0.3 M TritonX100, 0.1 M glycine in PBS), rinsed 2x in PBST (PBS, 0.1% Triton X100) and then incubated in blocking solution (0.1% Triton X-100, 1% v/v donkey serum in PBS) for 1 h at RT. Primary antibody incubation was carried out overnight at 4 °C. The next morning, the samples were washed 3x in PBST for 10 min at RT and then incubated in secondary antibodies plus DAPI in blocking solution for 2–3 h at RT or overnight at 4 °C in the dark. The embryos were washed 3x in PBST in the dark and then equilibrated through a glycerol series (25%/50% glycerol in PBST) for 15 min each at RT. The embryos were mounted in VECTASHIELD mounting medium (Vector Laboratories H-1200-10) between two cover slips to enable imaging from dorsal and ventral sides using vacuum grease and a 20x air objective on a Leica SP8 or a 20x air objective on a LSM800 or LSM 900 confocal microscope. For tile scans, 15% stitching overlap was used. The nucleoli of the germinal vesicles were imaged on a Nikon Eclipse Ti2 microscope equipped with a Yokagawa CSU W1 10,000 rpm Spinning Disk Confocal with 50 μm pinholes using a 100x oil objective.

Primary antibodies included: $\beta$-Catenin Thermo Fisher Scientific 71-2700, Lot GR184212-70 (rabbit, 1:200), Brachyury, R&D systems AF2085, Lot KQP0618021 (goat, 1:200), Laminin, Sigma-Aldrich L9393, Lot 099M4886V (rabbit, 1:100), pMyosinII, (rabbit, 1:100) 3761S, Lot 7.

Secondary antibodies (all 1:500) included: donkey-anti-rabbit-AF647 Life Technologies A31573, donkey-anti-goat-AF-488 Life Technologies A11055.

Stains included DAPI (1:500), Phalloidin-AF488 Life Technologies A12379 (1:500), Wheat Germ Agglutinin-AF555 Life Technologies W32464 (1:400).

### Histology

Embryos that had been previously stored in 100% MeOH were stepwise rehydrated through a descending methanol series (100/70/50/30 5 min each at RT), then dehydrated through an ascending methanol series to 70% EtOH (30/50/70 5 min each at RT). A couple of drops of Eosin were added to the 70% ethanol to lightly dye the embryos, which aids in orientation at embedding. Embryos were paraffin processed (Milestone, Pathos Delta Microwave Tissue Processor) using the following protocol w/o pressure: 70% EtOH 4 min at RT, 100% EtOH 5 min at 37 C, Isopropyl Alcohol 5 min at 45 C, Paraffin 15 min at 62 C. After processing, embryos were embedded in paraffin wax (Cancer Diagnostics, PureAffin® R56) and sectioned at a 5 µm thickness on a microtome (Leica RM2255). H&E staining was performed using an automatic stainer (DP360, Dakewe (Shenzen) Medical Equipment Co.) with ST Infinity H&E Reagents (Leica Biosystems Cat. 3801698). Slides were mounted with Cytoseal 60 mounting media (VWR, 48212-187). Embryos were imaged using an Olympus Slide Scanner on a 20x objective.

### RNA in situ hybridisation probes

Probes for *Lefty*, *Nodal1*, *Nodal2*, and *Cerberus* have been published and validated previously^17^. To clone RNA in situ probes for *Bmp2,* *Brachyury*, *Wnt3a* and *Nr6a1*, we collected embryonic RNA from appropriate stages when the genes were expressed. We used SuperScript III First-Strand Synthesis System for RT-PCR (Invitrogen 18080-051) for cDNA synthesis, utilising 100 ng of RNA per reaction. We carried out both Oligo (dT) and randomer hexamers reactions. The two reactions were mixed, and that cocktail was used to clone gene fragments for RNA in situ hybridisation probes.

The *Bmp2* probe is 608 bp and was cloned into the pGEM-T Easy vector. The sequences flanking the probe are as follows: F 5’- TGCTGGACACCCGGCTGATG and R 5’- TCAGCCCTCCACCACCATTT. The *Brachyury* probe is 449bp with following primers: F 5'-CTGGACCCCAACGCCATGTA and R 5'- TCCATCATGTCTTTGTGATCACTTCTT. *Wnt3a* probe is 500 bp and was cloned into the Zero Blunt TOPO vector (Invitrogen 450245) using the following primers: F 5’- GCCATTGGCCATCAATATTCC and R 5’- GTCCTTCCAGCTTCGTTGT. The *Nr6a1* probe is 760 bp and was cloned into the Zero Blunt TOPO vector (Invitrogen 450245) using the following primers: F 5’- GCTGATCGAAGATGGGTACAA and R 5’- AGAAACACACACGGGAGAAG.

### RNA in situ hybridisation

RNA in situ hybridisation was performed using a protocol optimised for chameleon embryos^17^. All incubation steps, unless otherwise stated, were carried out on a rocker. In short, embryos were dehydrated through an ascending methanol series into 100% MeOH (25/50/75/100/100% 5 min each at RT) and stored overnight at −20 °C. The following day, the embryos were re-hydrated through a descending methanol series (75/50/25% MeOH 5 min each at RT) and washed 2x 5 min in PBST/DEPC. The embryos were treated for 7 min with proteinase K (10ug/ml) without shaking and then refixed in 4%(v/v) PFA/DEPC, 0.1% (v/v) glutaraldehyde for 20 min. The embryos were then rinsed 1x 2 min and washed 2x5min in PBST, and transferred to 50% PBST/DEPC 50% pre-hybridisation buffer (50% formamide, 2% SDS, 2% blocking reagent (all v/v), 5x SSC pH7.0, 250ug/ml tRNA, 100ug/ml Heparin) for 10 min at 68 °C. The embryos were then incubated 1 x 10min and 1 x 1 h in 100% pre-hybridisation buffer at 68 °C. The probes were added at 1 ug/ml in pre-hybridisation buffer, and the embryos were incubated overnight at 68 °C. Next day, the probes were removed and the embryos rinsed (2 min) in prewarmed solution X (50% (v/v) formamide, 1% (v/v) SDS, 2x SSC pH7.0) followed by 4 washes in solution X for 30 min each at 68 °C. Then, embryos were washed in solution X:MABT (1:1) for 10 min at 68 °C and then moved to RT for all following steps. The embryos were rinsed 3x in MABT (2 min) and washed 2x 30 min) and then washed 1 x 1 h in MABT + 2% (w/v) blocking reagent, followed by 1x1h washed in 20% (v/v) lamb serum, 2% (w/v) blocking reagent in MABT. Anti-DIG was added in 20% (v/v) lamb serum, 2% (w/v) blocking reagent in MABT at 4 °C overnight. The next day, the embryos were washed 3x5min in MABT, 7 x 45 min in MABT at RT, followed by 4 x 10 min washes in NTMT (100 mM NaCl, 100 mM Tris pH9,5, 50 mM MgCl2, 1% (v/v) Tween2-, 2 mM levasimole). The colour reaction was then developed in the dark at RT in NTMT + 1;400 BCIP & NBT each). The reaction was stopped at the desired intensity with PBST. The embryos were then refixed in 4% PFA and stored at 4 °C. For each time point, a sense control was run in parallel to the antisense probes.

The probes for *Lefty*, *Nodal1*, *Nodal2* and *Cerberus* have been published and validated previously^17^. The probes for *Brachyury*, *Bmp2*, *Nr6a1* and *Wnt3a* were newly prepared for this study.

### Statistics and reproducibility

For each experimental parameter, between 3–4 embryos were analysed per female. The embryos of an individual clutch are roughly time-matched but not as identical as found for embryos from a mouse litter. As such, embryos from one female may be distributed across 2–3 developmental stages. For H&E staining, embryos of consecutive stages were assessed. For in situ hybridisation experiments, embryos from 8–10 individual females were analysed depending on the probe. For immunofluorescence staining, when focussing on a specific stage, embryos of 1–2 different females were stained. When immunofluorescence staining focused on consecutive stages, embryos of 2–5 females were utilised, depending on how many stages were assessed.

### Scanning electron microscopy

Samples for scanning electron microscopy were prepared as previously described^30^. Briefly, after fixation, samples were processed using a TOTO method (tannic acid, osmium tetroxide, thiocarbohydrazide, and osmium tetroxide), dehydrated in a graded series of ethanol, and dried in a Tousimis Samdri 795 critical point dryer. After mounting on stubs, samples were imaged in a Zeiss Merlin SEM at 8 kV using the SE2 detector.

### Transmission electron microscopy

Samples for TEM imaging were also prepared as previously described, with secondary fixation with osmium tetroxide and en bloc staining with uranyl acetate before dehydration in a graded series of ethanol, propylene oxide as a transitional solvent, and infiltration with Hard Plus resin (Electron Microscopy Sciences). After embedding, samples were cured at 60 °C, and then samples in blocks were imaged in a Bruker SkyScan 1272 microCT to identify regions of interest, sectioned at 80 nm with a Diatome diamond knife and imaged in a Zeiss Merlin SEM at 26 kV and 700 pA with a STEM detector.

### Brightfield imaging

Embryos were placed in a petri dish coated with Agarose and filled with PBS. Imaging was carried out on a Leica DFC550 microscope. Manual stacks were taken that were rendered using the Helicon software (Focus 5.3 software).

### Image processing

SP8

The images shown in Figs. 1F, S4B were performed using bidirectional scanning, which resulted in every odd scan-line being shifted by 4 pixels. This happened due to a misalignment of the two scanners. The images were then corrected computationally by shifting every odd scan-line by 4 pixels to align with the even lines. The simple correction was applied in Python.

### Image analysis

All image analysis was carried out in Fiji, and all graphs and statistics were prepared using Graphpad Prism 10 & 11. For all measurements, the ROIs were saved to enable traceability of the measurements.

### Reporting summary

Further information on research design is available in the Nature Portfolio Reporting Summary linked to this article.

## Supplementary information

Supplementary Information

Peer Review file

Reporting Summary

## Source data

Source Data 1

Source Data 2

Source Data 3

Source Data 4

## Data Availability

Upon publication, the original data underlying this manuscript can be accessed from the Stowers Original Data Repository at https://www.stowers.org/research/publications/libpb-2622.

## Code availability

No novel code has been generated for this study.
