# Supplementary material for: Epiblast lumenogenesis is not a mammalian-specific trait
Source: Nat Commun. 2026 Jun 3;17:7106. doi: 10.1038/s41467-026-73768-9 (PMC13392392; doi:10.1038/s41467-026-73768-9)
Supplement: Supplementary file 1 — Supplementary Information [file 41467_2026_73768_MOESM1_ESM.pdf]

Supplementary Information

Figure S1

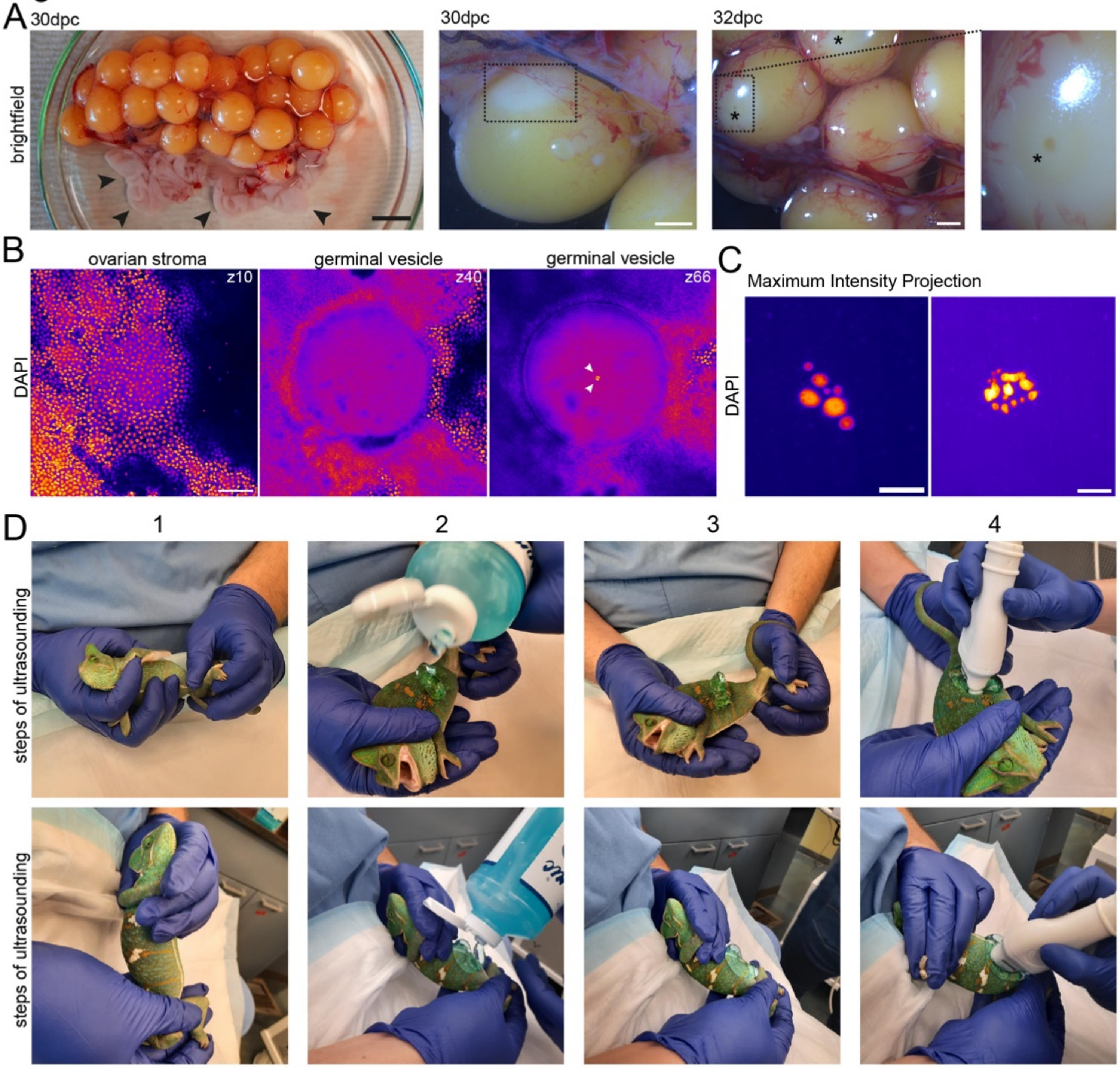

A
30dpc
brightfield
30dpc
32dpc
*
*
*
B
ovarian stroma
germinal vesicle
germinal vesicle
z10
z40
z66
DAPI
C
Maximum Intensity Projection
DAPI
D
1
2
3
4
steps of ultrasounding
steps of ultrasounding

Figure S1: Oocyte morphology of the veiled chameleon.
A. Brightfield imaging. Left: ovary filled with vitellogenic follicles at 30dpc. The uterine horn is visible beneath the ovary (arrows). middle/right: mature vitellogenic follicles inside the ovary with germinal discs (square) and germinal vesicles visible (asterisks) at 30dpc(middle) and 32dpc (right). representative of n=3. Scale bars left 2cm, middle/right 2mm. **B.** Further examples of confocal imaging of DAPI stained germinal discs. Z-stack through the germinal vesicle, z10/40/66 are shown. The middle of the germinal vesicle is DAPI positive, arrows highlight DAPI-positive region. Scale bar 100μm. **C.** Further examples of maximum intensity projection of spinning disc imaging of DAPI-positive centre of 2 germinal vesicles. DNA has segregated into distinct foci. Scale bars 10μm. **D.** Chameleon handling for ultrasound of abdominal cavity.

Figure S2

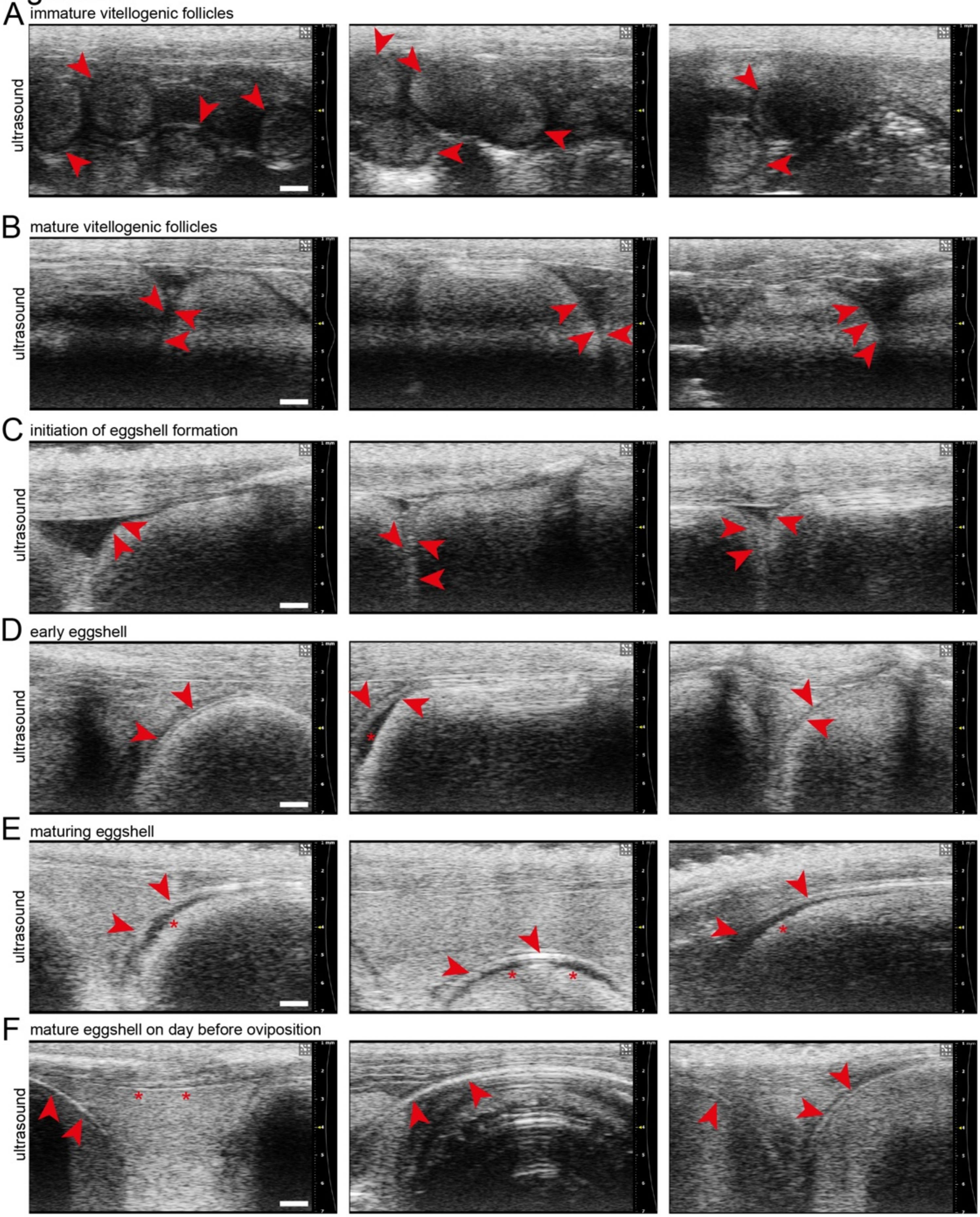
A immature vitellogenic follicles
B mature vitellogenic follicles
C initiation of eggshell formation
D early eggshell
E maturing eggshell
F mature eggshell on day before oviposition
ultrasound

Figure S2: Following folliculogenesis and eggshell maturation via ultrasound
A-F. Ultrasound imaging of veiled chameleon abdomen. **A.** immature vitellogenic follicles arrows indicate follicles. **B.** mature vitellogenic follicles. Arrows indicate border between follicles which is dark indicating no shell development yet. **C.** initiation of eggshell formation. arrows indicate the thin eggshells in-between eggs. **D.** early eggshell. Arrows indicate eggshell, asterisks point out space between yolk and eggshell characteristic for early shelling stages. **E.** maturing eggshell. Arrows indicate thickened eggshell, asterisks the space between yolk and shell. **F.** mature eggshell on day before oviposition. Arrows indicate eggshell, no empty space between yolk and eggshells at mature stages. asterisks indicate uterine wall. All scale bars 1mm. ultrasound performed for 12 females.

Figure S3

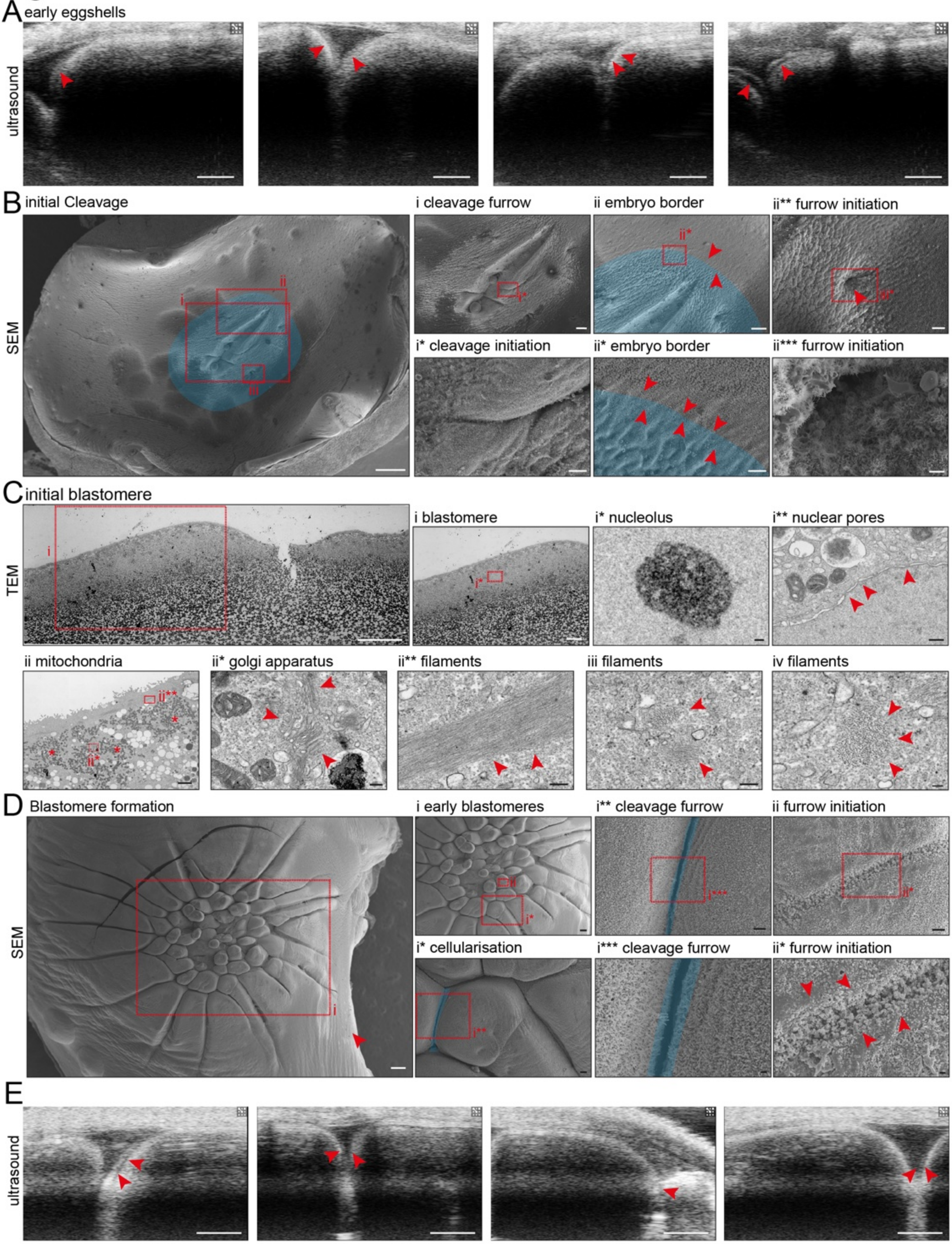
A early eggshells
ultrasound
B initial Cleavage
SEM
i cleavage furrow
ii embryo border
ii** furrow initiation
i* cleavage initiation
ii* embryo border
ii*** furrow initiation
C initial blastomere
TEM
i blastomere
i* nucleolus
i** nuclear pores
ii mitochondria
ii* golgi apparatus
ii** filaments
iii filaments
iv filaments
D Blastomere formation
SEM
i early blastomeres
i** cleavage furrow
ii furrow initiation
i* cellularisation
i*** cleavage furrow
ii* furrow initiation
E
ultrasound

Figure S3: Initial cleavage division pattern

A. Ultrasound imaging of eggshells of the eggs dissected for Figure 2B-C, SB/C. arrows indicate the very thin eggshells. scale bars 2mm. **B.** SEM imaging of initial embryo cleavage. blue highlight indicates area of embryonic plate. Squares indicates regions of zoom-ins. (i) cleavage furrow. Zoom-in on initial cleavage (i*) cleavage initiation. (ii/ii*) embryo border. Blue indicates embryonic plate, arrows point out embryo border. (ii**/***) furrow initiation. arrow indicated hole that facilitates cleavage initiation at the embryo border. Scale bars: initial cleavage=400μm, i/ii=100μm, i*/ii*=20μm, ii**=40μm, ii***=5um. **C.** TEM of initial cleavage stage blastomere. Squares indicated regions of zoom-ins. (i) blastomere localisation. (i*) zoom-in on nucleolus. (i**) nuclear pores. Arrows indicates nuclear pores. (ii) mitochondria. Asterisks indicate regions of mitochondria. Squares indicate regions of zoom-in. (ii*) Golgi apparatus. Arrows indicate Golgi. (ii**/iii-iv) filaments. Arrows indicate differently-angled filaments in cytoplasm. scale bars: initial blastomere=100μm, i=40μm, i*/iv=100nm, i**=300nm, ii=3um, ii*/**/iii=200nm. **D.** SEM of blastomere formation. Squares indicate regions of zoom-ins (i) early blastomeres in middle of embryonic plate. (i*) cellularisation. Blue highlight mature cleavage furrow. (i**/***) cleavage furrow. Furrow highlighted in blue. (ii) furrow initiation. arrows indicate initiating furrow. scale bars: blastomere formation=200μm, i=100μm, i*=20μm, i**/ii=10μm, i***/ii*=2um. **E.** Ultrasound imaging of eggshells of eggs dissected for Figure 2D/S3D). arrows indicate eggshell. all scale bars=2mm.

Figure S4

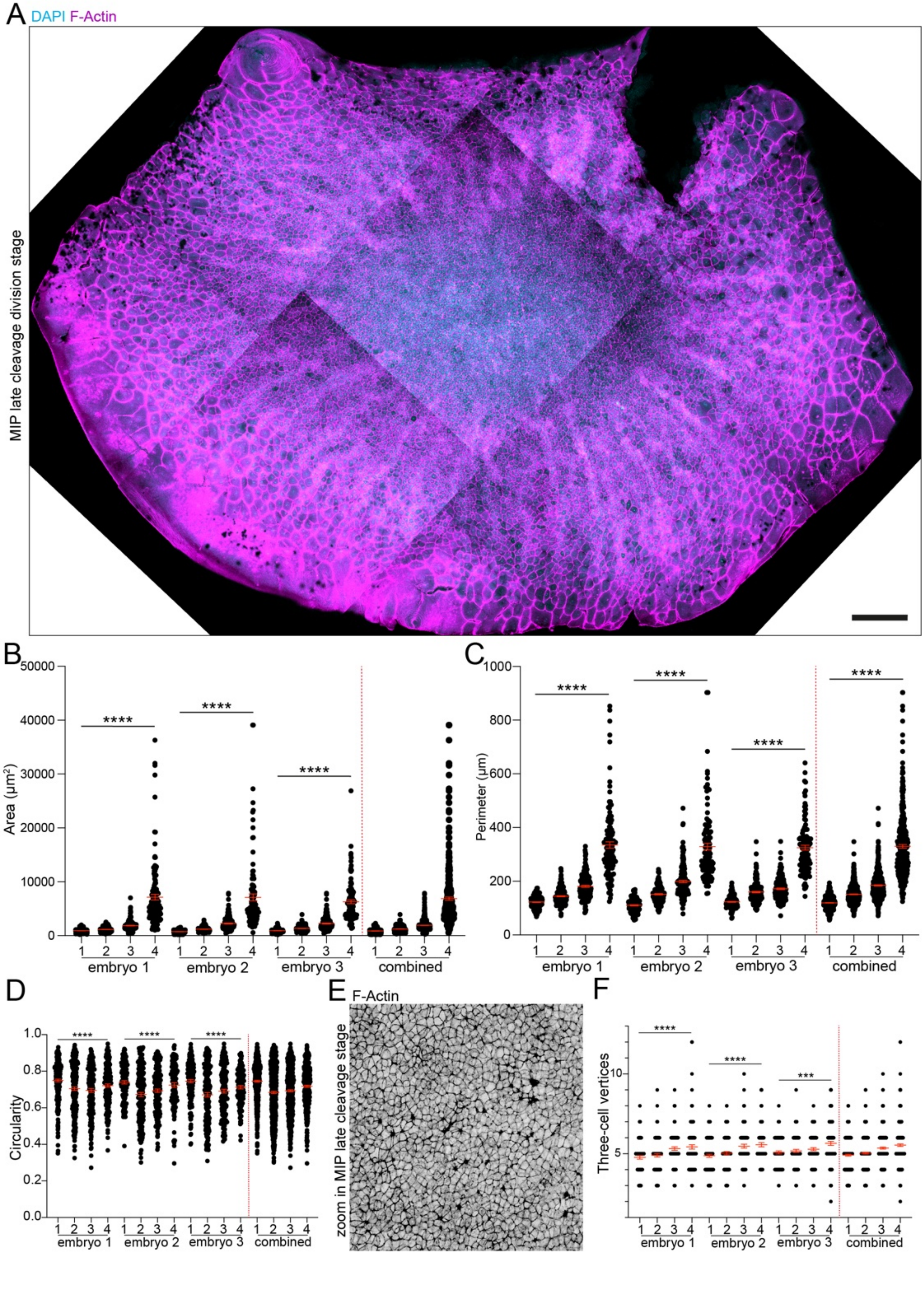
A DAPI F-Actin
MIP late cleavage division stage
B
Area (µm²)
50000
40000
30000
20000
10000
0
****
1 2 3 4
embryo 1
embryo 2
embryo 3
combined
C
Perimeter (µm)
1000
800
600
400
200
0
D
Circularity
1.0
0.8
0.6
0.4
0.2
0.0
E F-Actin
zoom in MIP late cleavage stage
F
Three-cell vertices
10
5
***

Figure S4: Quantitative analysis of cell shapes during late cleavage stages.
A. maximum intensity projection of late cleavage stage embryo. dorsal view. DAPI (cyan), F-Actin (magenta). replicate to Figure 2E. Scale bar 500um. **B.** Analysis surface area of embryonic cells of areas 1-4. Analysis of 3 embryos per area. Combined plot shown in Figure 2H. Scatter plot plus mean±SEM. Statistical Analysis One-way Anovas for each embryo p-value<0.0001. unpaired t-tests for each embryo area 1-2/2-3/3-4 p-value<0.0001. statistical analysis for combined data in main figure 2H. **C.** Analysis surface perimeter of embryonic cells of areas 1-4. Analysis of 3 embryos per area. Combined data on the right. Scatter plot plus mean±SEM. Statistical Analysis One-way Anovas for each embryo p-value<0.0001. Unpaired two-sided t-tests for each embryo and combined embryos area 1-2/2-3/3-4 p-value<0.0001 except for embryo 3 area2-3, here p-value 0.0039. **D.** Analysis cell circularity of embryonic cells of areas 1-4. Analysis of 3 embryos per area. Combined plot shown in Figure 2I. Scatter plot plus mean±SEM. Statistical Analysis One-way Anovas for each embryo p-value<0.0001. unpaired two-sided t-tests for each embryo area 1-2/2-3/3-4. P-values: Embryo 1 (1-2) =0.0003, (2-3)=0.4937, (3-4)=0.0563. Embryo 2 (1-2)<0.0001, (2-3)=0.1647, (3-4)=0.0434. Embryo 3 (1-2)<0.0001, (2-3)=0.1985, (3-4)=0.1542. statistical analysis for combined data in main figure 2I. **E.** Rosettes within the centre of late cleavage stage embryos. Unlabelled image of Figure 2J. **F.** Analysis three-cell vertices of areas 1-4. Analysis of 3 embryos per area. Combined plot shown in Figure 2K. Scatter plot plus mean±SEM. Statistical Analysis One-way Anovas for embryo 1/2 p-value<0.0001, embryo 3 p-value=0.0007. Unpaired two-sided t-tests for each embryo area 1-2/2-3/3-4. p-values: Embryo 1 (1-2)=0.4308, (2-3)=0.0057, (3-4)=0.5422. Embryo 2 (1-2)=0.2555, (2-3)=0.0039, (3-4)=0.6229. Embryo 3 (1-2)=0.4645, (2-3)=0.4330, (3-4)=0.0211.

Figure S5 - stages of embryogenesis

Stage		Description
Stage I	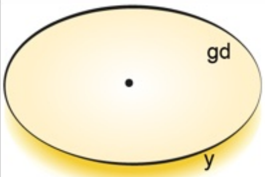	**Fertilisation**<br>germinal disc (gd)white on top of yolk (y)
Stage II	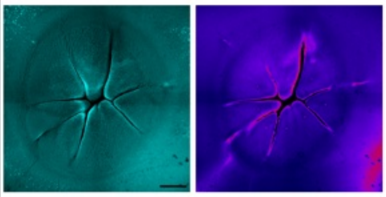	**initial Cleavage**<br>radial pattern of initial cleavage furrows<br>distinct structure of embryonic plate within germinal disc
Stage III	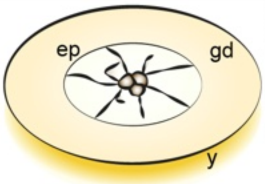	**Cellularisation**<br>first blastomeres form in centre<br>embryonic plate extends<br>cleavage furrows to edge of embryonic plate
Stage IV	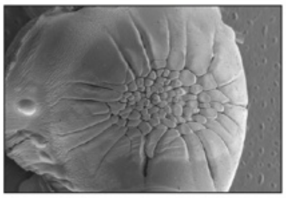	**Cellularisation**<br>middle of embryonic plate filled with blastomeres<br>plate extends over entire germinal disc
Stage V	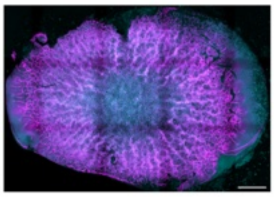	**Subcleavages**<br>entire ep filled with blastomeres<br>small blastomeres in middle, large, open outside<br>large furrows from centre to edge
Stage VI	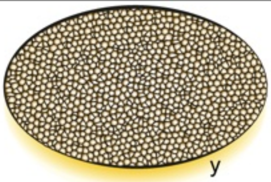	**Cleavage completion**<br>entire embryonic plate filled with small blastomeres<br>blastomeres not adherent yet
Stage VII	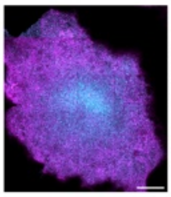 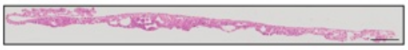	**epiblast plate**<br>epiblast flat, tightly packed adherent disc<br>hypoblast as spongy tissue beneath, thicker in middle of embryo
Stage VIII	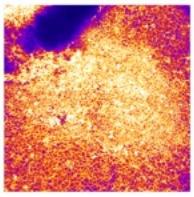 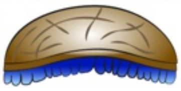	**Dome initiation**<br>epiblast initiates curvature<br>central cells smaller than outer cells
Stage IX	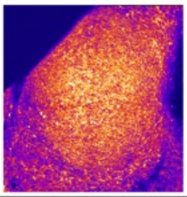 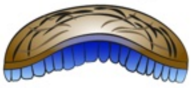	**cell alignment**<br>epiblast dome increases<br>side cells align long axis with concentric rings surrounding epiblast centre
Stage X	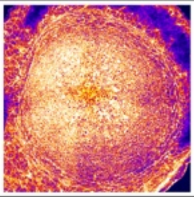	**tension increase**<br>epiblast highly domed<br>side cells thinly stretched and start exhibiting higher tension
Stage XI	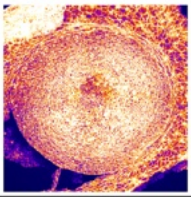	**Hinge Points**<br>epiblast highly domed<br>concentric supracellular actin cables<br>amnion folds initiate as small shoulders
Stage XII	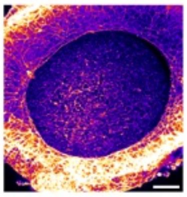	**Purse string I**<br>amnion folds start to elevate & constrict forming wide ring around epiblast
Stage XIII	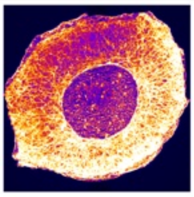 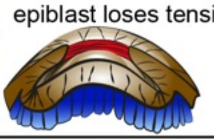	**Purse-string II**<br>amnion folds constrict, high dorsal tension<br>epiblast loses tension
Stage XIV	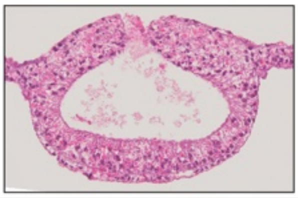	**Fold apposition**<br>amnion folds appose<br>actin cable constricted to small opening<br>squamouse trophoblast-like layer overlays folds
Stage XV	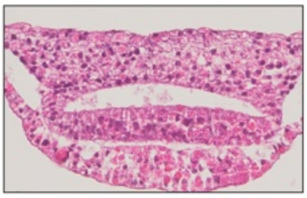	**Lumenogenesis complete**<br>radially symmetric embryo<br>closure point visible as slight indentation
Stage XVI	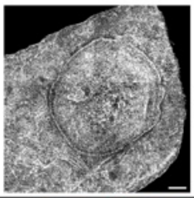	**AP-axis initiation**<br>embryo broadens<br>AP-markers define axis
Stage XVII	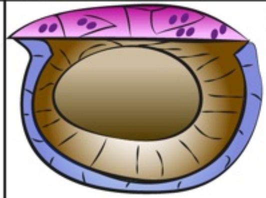	**AP-axis completion**<br>anterior side thinner<br>posterior side thickened
Stage XVIII	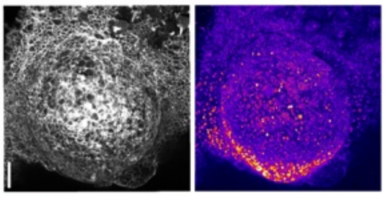	**Initiation gastrulation**<br>cells in posterior initiate EMT<br>anterior epiblast much thinner
Stage XIX	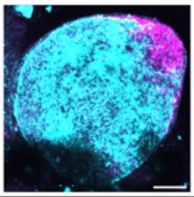 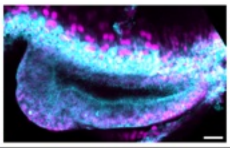	**Mesoderm ingression initiation**<br>breach of basement membrane<br>mesoderm initiates i ngression

Figure S5: The stages of chameleon pre-oviposition development
Table with images or schematics depicting each stage of development accompanied by short descriptions

Figure S6

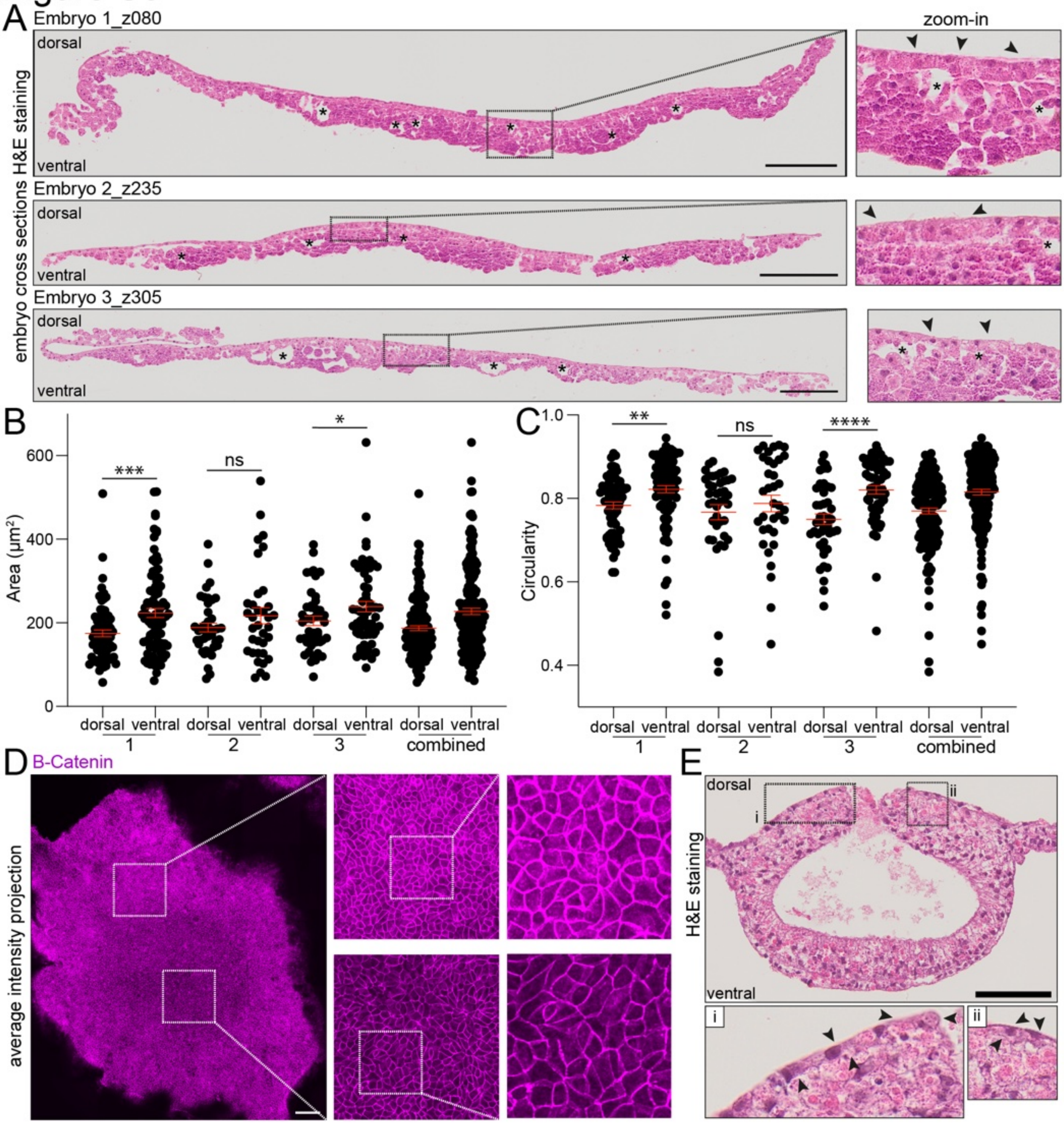
A
Embryo 1_z080
zoom-in
dorsal
ventral
H&E staining
embryo cross sections
Embryo 2_z235
dorsal
ventral
Embryo 3_z305
dorsal
ventral
B
Area (μm²)
0
200
400
600
***
ns
*
dorsal ventral
1
dorsal ventral
2
dorsal ventral
3
dorsal ventral
combined
C
Circularity
1.0
0.8
0.6
0.4
**
ns
****
dorsal ventral
1
dorsal ventral
2
dorsal ventral
3
dorsal ventral
combined
D
B-Catenin
average intensity projection
E
dorsal
ventral
H&E staining
i
ii

Figure S6: Analysis of cell shapes in embryos following cell-cell adhesion

A. H&E staining of cross sections of 3 embryos following cell-cell adhesion. squares indicate regions of zoom-in. asterisks indicate holes in the hypoblast layer. arrows indicate epiblast layer. All scale bars:200µm. **B/C.** Quantitative analysis of dorsal to ventral cell populations of embryos shown in (A). (B) Cell area. Scatter plot plus mean±SEM. Statistical Analysis two-sided unpaired t-tests dorsal-ventral p-values (1)=0.0007, (2)=0.1887, (3)=0.0500. (C) Cell circularity. Scatter plot plus mean±SEM. Statistical Analysis two-sided unpaired t-tests dorsal-ventral p-values (1)=0.0016, (2)=0.4391, (3)$<$0.0001. **D.** Immunofluorescence staining of β-Catenin in embryo post cell-cell adhesion, maximum intensity projection. Squares indicate areas of zoom-in. Scale bar=200µm. **E.** H&E staining of embryo during lumenogenesis stages, non-pseudo-coloured image of Figure 3G. squares indicate region of zoom-in. emerging trophoblast-like layer annotated with arrows. scale bar 100µm.

Figure S7

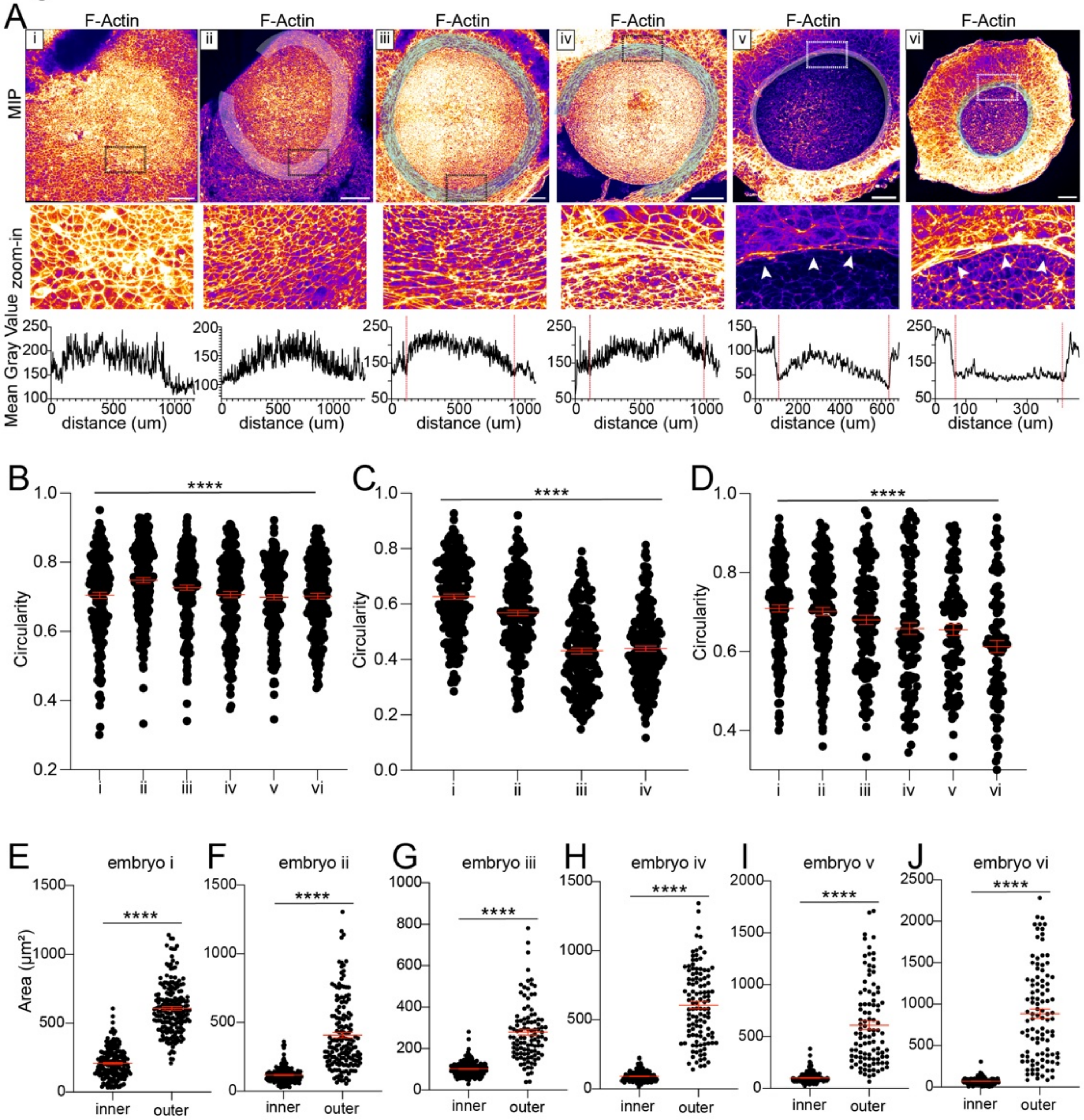
A
F-Actin
i
ii
iii
iv
v
vi
MIP
zoom-in
Mean Gray Value
distance (um)
B
C
D
Circularity
****
E
embryo i
F
embryo ii
G
embryo iii
H
embryo iv
I
embryo v
J
embryo vi
Area (μm²)
inner
outer

Figure S7: Analysis of Cell shapes during Lumenogenesis

A. top row maximum intensity projections of F-actin during lumenogenesis (i-vi) in whole embryos. squares indicate zoom-in regions of the second row. white/blue highlights indicate epiblast border. Columns v/vi arrows indicate supracellular actin cable. Bottom row. Plot profiles of actin mean gray value drawn across the length of the embryo. red lines indicate border between epiblast and fold/outer cells. Fire staining (yellow-white: high signal intensity, purple-black low signal intensity). Scale bars: (i-iv)=200μm, (v-vi)= 100μm. 6 consecutive embryos. **B-D.** Quantitative Analysis of Cell Circularity during lumenogenesis (embryos i-vi) in (A) central cells, (B) cells in ring surrounding epiblast centre, (C) outer cells. Scatter plot plus mean±SEM. Statistical Analysis One-way Anovas, all p-values<0.0001. **E-J.** Quantitative analysis of cell area in middle (inner) versus peripheral (outer) cells during lumenogenesis stages i-vi (corresponding to D-I). Scatter plot plus mean±SEM. Statistical Analysis One-way Anovas, all p-values<0.0001.

Figure S8

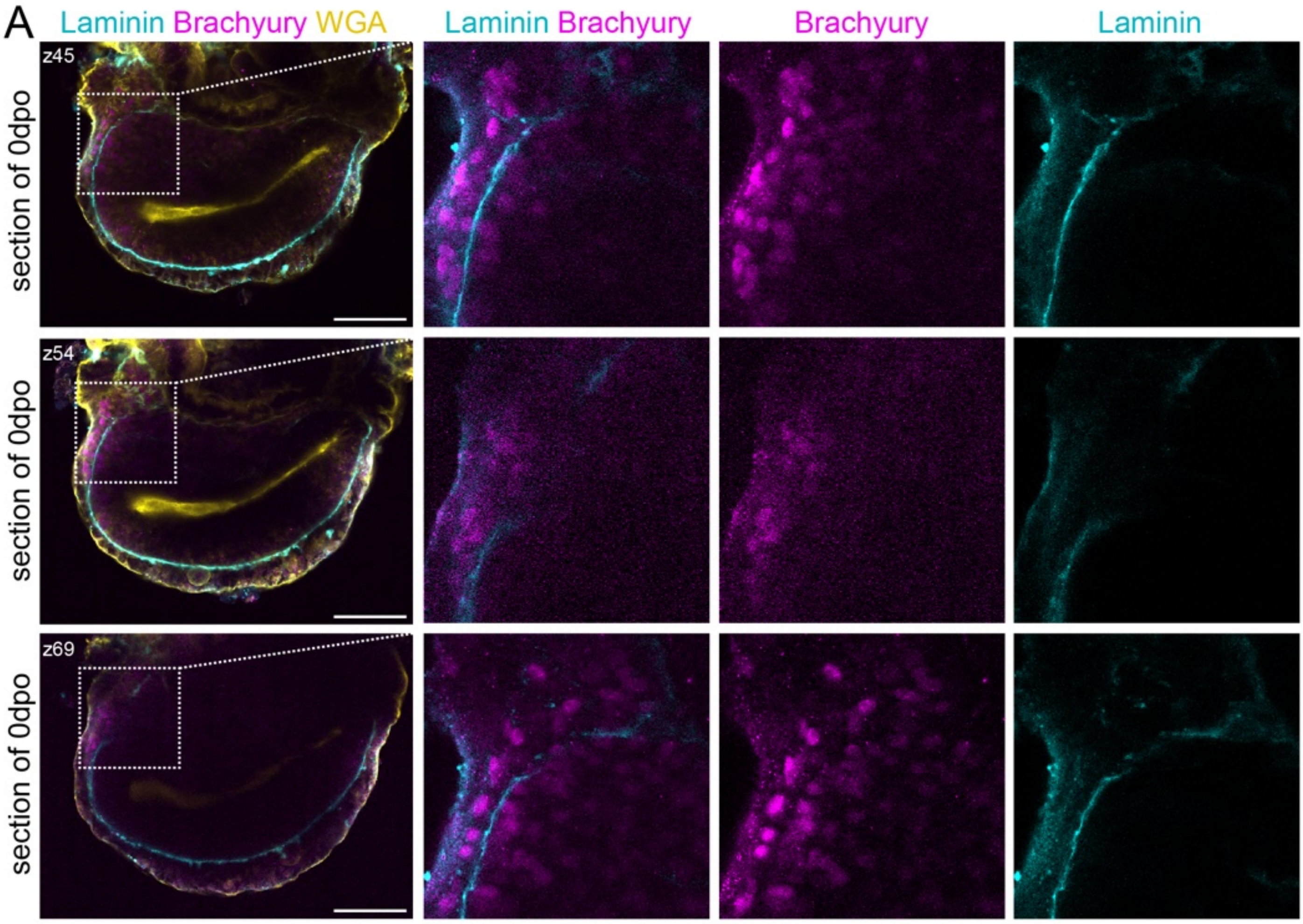
A
Laminin Brachyury WGA
Laminin Brachyury
Brachyury
Laminin
z45
section of 0dpo
z54
section of 0dpo
z69
section of 0dpo

Figure S8: Cell ingression during gastrulation

A. virtual cross sections of 0dpo gastrulating embryo. row1-3 different cross sections. Laminin (cyan), Brachyury (magenta), WGA (yellow). squares indicate zoom in. All scale bars 100um.

Figure S9

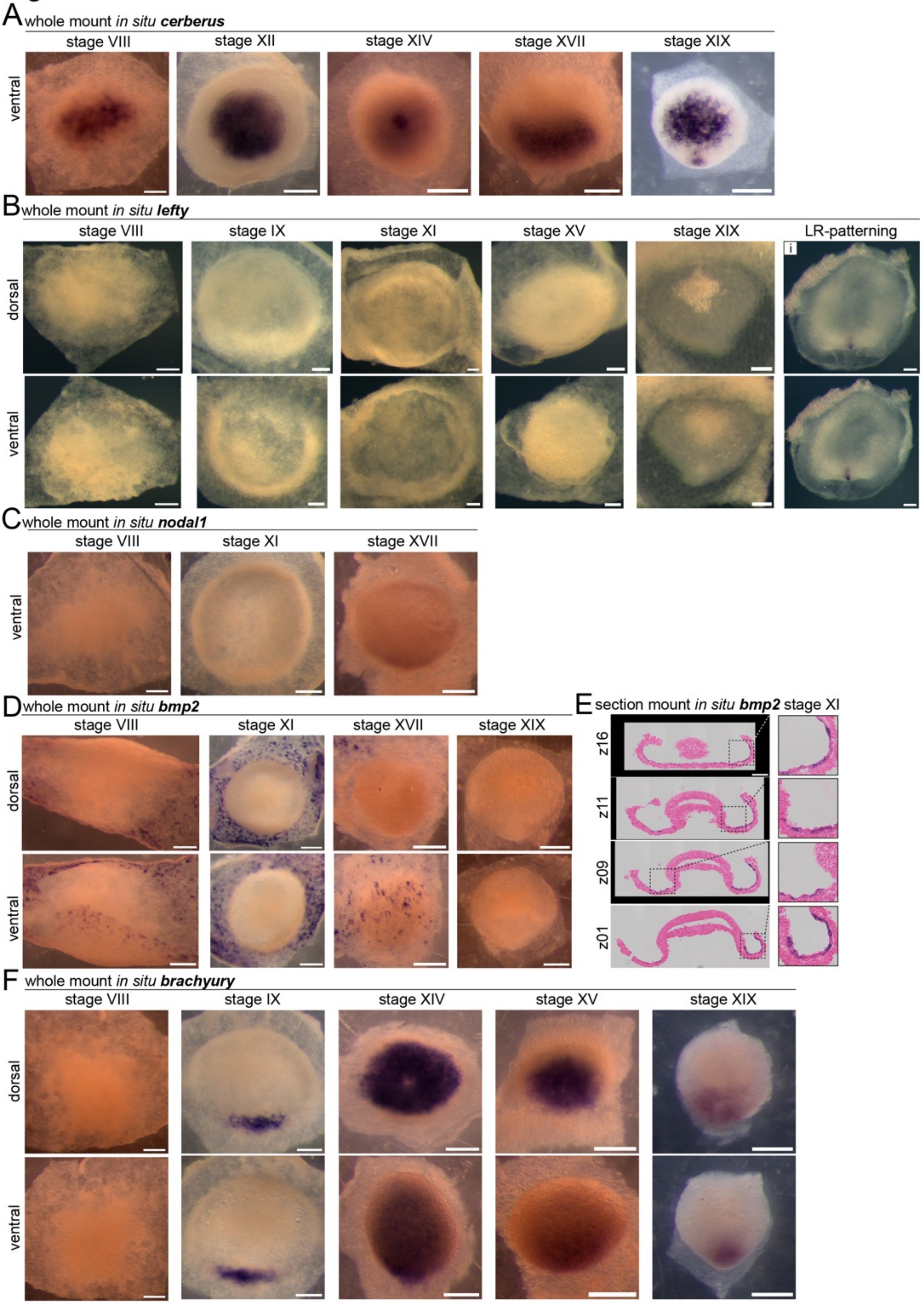

A whole mount in situ cerberus
stage VIII
stage XII
stage XIV
stage XVII
stage XIX
ventral
B whole mount in situ lefty
stage VIII
stage IX
stage XI
stage XV
stage XIX
LR-patterning
i
dorsal
ventral
C whole mount in situ nodal1
stage VIII
stage XI
stage XVII
ventral
D whole mount in situ bmp2
stage VIII
stage XI
stage XVII
stage XIX
dorsal
ventral
E section mount in situ bmp2 stage XI
z16
z11
z09
z01
F whole mount in situ brachyury
stage VIII
stage IX
stage XIV
stage XV
stage XIX
dorsal
ventral

Figure S9: Expression patterns of anterior-posterior patterning and gastrulation regulators.

A-E. further examples of whole mount in situ hybridisations at subsequent stages of development from stage VIII-XIX of (**A**) *Cerberus*, (**B**) *Lefty*, (**C**) *Nodal1*, (**D**) *Bmp2*, (**E**) cross section of *Bmp2* staining at stage XI. Squares indicate regions of zoom-in. **(F)** *Brachyury*. Stages are annotated on top of each column. All scale bars 200μm.

Figure S10

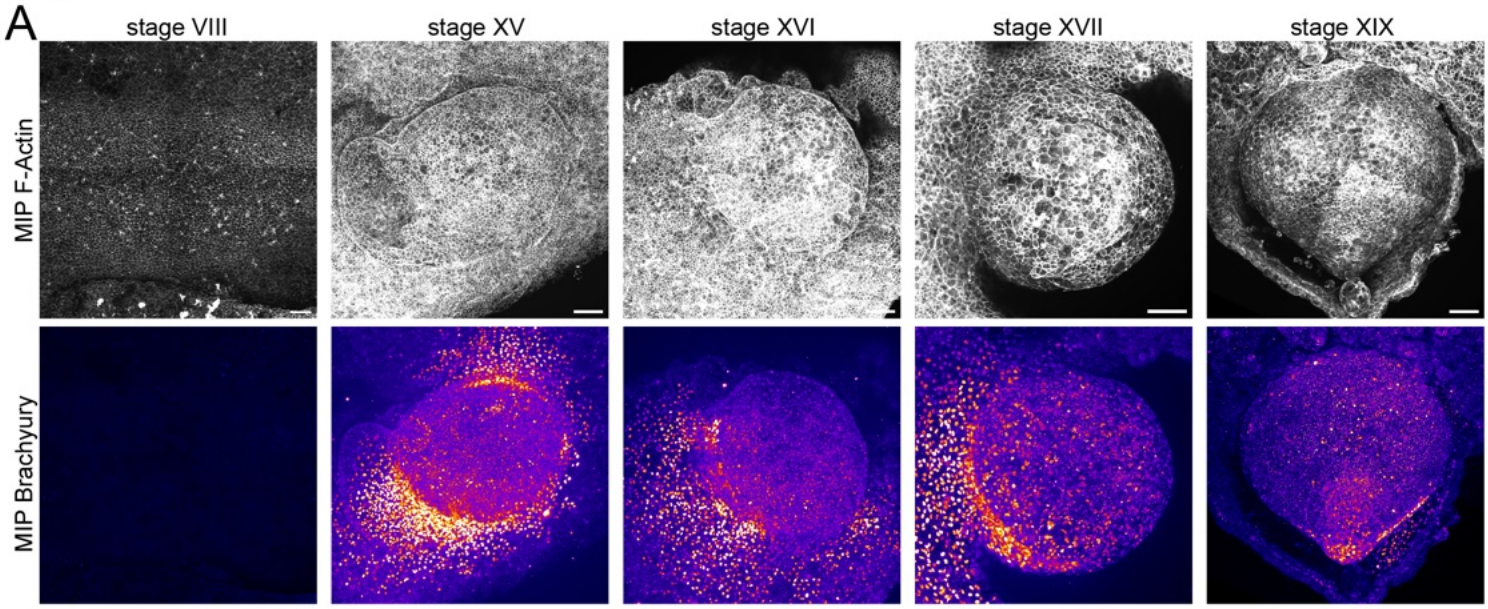
A
stage VIII
stage XV
stage XVI
stage XVII
stage XIX
MIP F-Actin
MIP Brachyury

Figure S10: Brachyury protein expression patterns

A. Further examples of maximum intensity projection of F-actin (top row) and Brachyury (bottom row) shown in Figure 6C/D. Embryo stages defined on top of each column. All scale bars 100µm.
