# Supplementary figures and images for: Epiblast lumenogenesis is not a mammalian-specific trait
Source: Nat Commun. 2026 Jun 3;17:7106. doi: 10.1038/s41467-026-73768-9 (PMC13392392; doi:10.1038/s41467-026-73768-9)

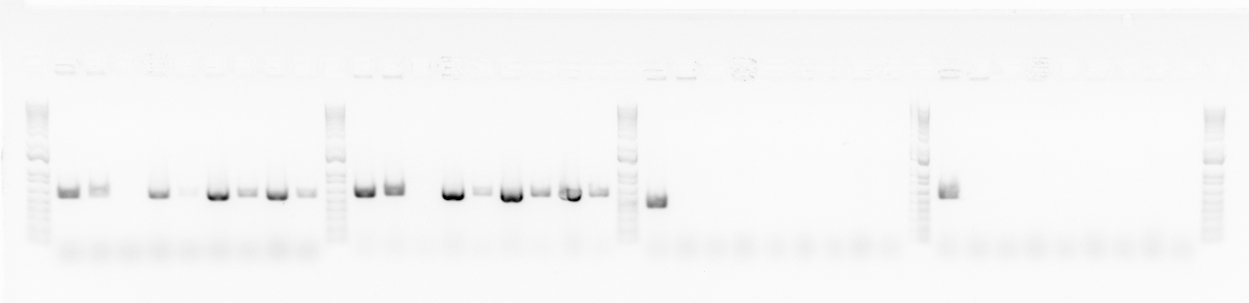

Supplement: Supplementary file 5 — Source Data 2 [file 41467_2026_73768_MOESM5_ESM.zip › SourceData_Figure 1/SourceData_Figure 1H-I_whole gel_20240127_full_0.625sec.jpg]

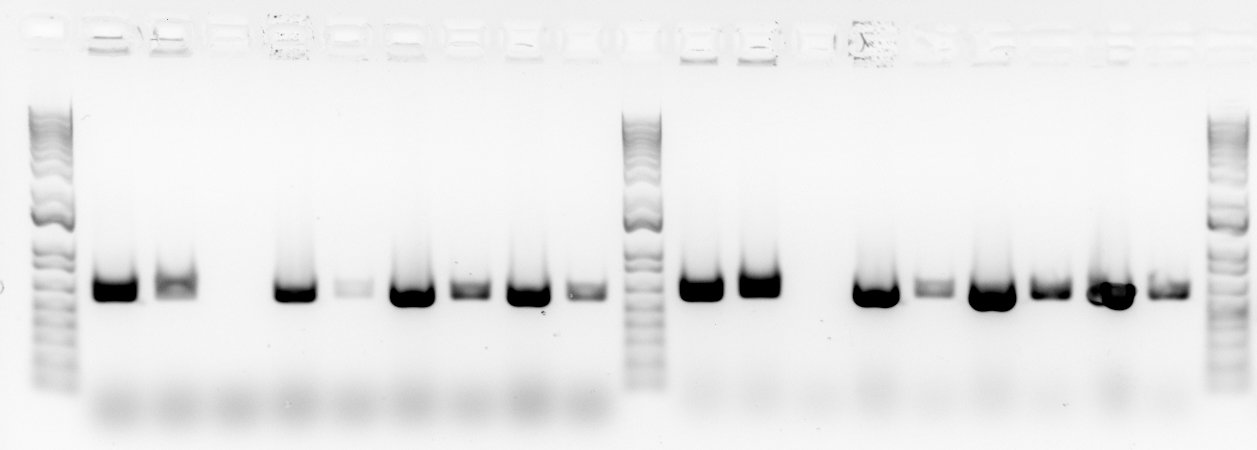

Supplement: Supplementary file 5 — Source Data 2 [file 41467_2026_73768_MOESM5_ESM.zip › SourceData_Figure 1/SourceData_Figure 1H_20240127_Hox8-9_1.25sec.jpg]

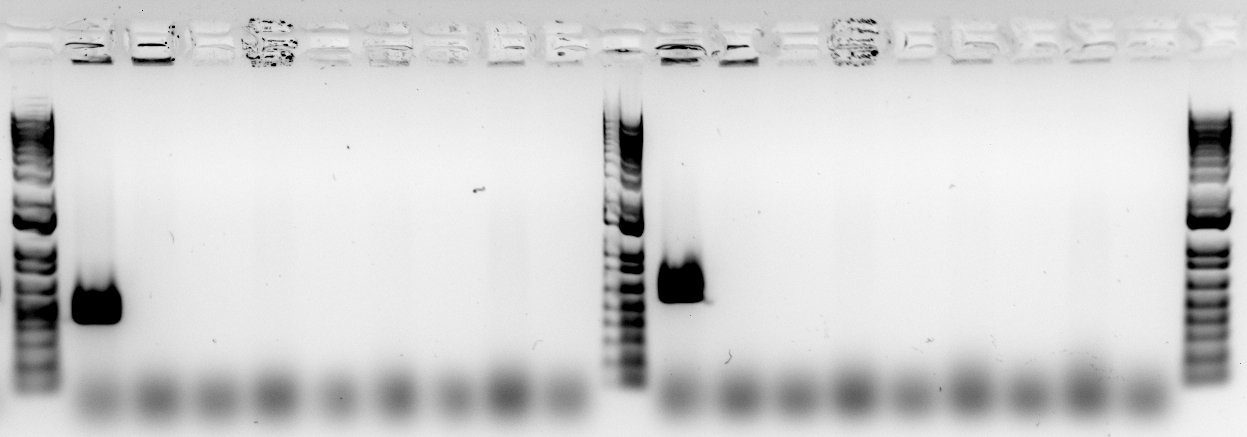

Supplement: Supplementary file 5 — Source Data 2 [file 41467_2026_73768_MOESM5_ESM.zip › SourceData_Figure 1/SourceData_Figure 1I_20240127_M2-3_4sec.jpg]
