# Supplementary material for: Epiblast lumenogenesis is not a mammalian-specific trait
Source: Nat Commun. 2026 Jun 3;17:7106. doi: 10.1038/s41467-026-73768-9 (PMC13392392; doi:10.1038/s41467-026-73768-9)
Supplement: Supplementary file 2 — Peer Review file [file 41467_2026_73768_MOESM2_ESM.pdf]

nature portfolio

Peer Review File

Epiblast lumenogenesis is not a mammalian-specific trait

Corresponding Author: Dr Antonia Weberling

This file contains all reviewer reports in order by version, followed by all author rebuttals in order by version.

Version 0:

Reviewer comments:

Reviewer #1

(Remarks to the Author)
Here, Weberling and colleagues examine development of the veiled chameleon before oviposition, including lumen formation in the epiblast and subsequent gastrulation. The study begins with a detailed description of chameleon development, including early embryonic cleavages and cellularization. Through careful analysis of staged embryos, they then describe the formation of tissue folds at the edge of the epiblast that elevate dorsally and then close via a purse-string mechanism to enclose an amniotic cavity with a ventral epiblast and dorsal amnion. Soon after the epiblast lumen closes, the embryo breaks anteroposterior symmetry and mesoderm cells are seen ingressing at the posterior end. This ingression coincides with breakdown of the basement membrane and brachyury expression is observed in the nascent mesoderm, but brachyury expression is first observed prior to ingression. It is initially expressed radially in the future amnion before resolving to the site of gastrulation, exhibiting distinct patterns from other amniotes. The authors characterize mRNA expression of additional signaling molecules, with some exhibiting patterns expected from other species (Nodals) and others show novel expression patterns (Wnt3). This study beautifully describes key developmental stages of an emerging model organism, including staging criteria, providing a valuable resource for the community. It further highlights exciting differences and similarities between reptile, avian, and mammalian embryogenesis that provide key insights into the evolution of development. Some statements were identified that require clarification or additional supportive evidence in this otherwise strong study, which are listed below.

1. In the abstract, the authors state that AP patterning occurs independently of Cerberus and Lefty. While the expression patterns of these genes suggest this is likely true, no functional evidence is provided in support of it. I recommend revising this statement accordingly.

2. The authors repeatedly make claims about tissue tension based on actin staining patterns. I would agree that these patterns are suggestive of increased tension, but not indicative. (Functional tests of tissue recoil after laser ablation, etc would be necessary to make these claims). I recommend revising such statements accordingly.

3. The authors report the appearance of spindle-shaped ingressing/ ingressed mesoderm cells in the posterior epiblast, but their shape nor their identity is apparent from the EM image shown in Fig. 4B. These cells are much more clearly shown in panels (Fig. 4E) depicting immunofluorescent staining for Brachyury, but their location is not consistent with those outlined in 4B. 4B shows these cells dorsally near the border with the amnion, while they appear more ventrally located in 4E and seem to protrude from the epiblast. Can the authors reconcile these apparently distinct descriptions of the nascent mesoderm?

4. The expression patterns of Brachyury described and shown in fig 5G-H are very interesting, but as presented are somewhat confusing. 5H shows 2 dorsal expression domains at stage XIX, while wholemount staining suggests there should be a single ventral patch at the posterior site of gastrulation. Is the stage of some images labeled incorrectly?

5. When discussing Brachyury protein expression patterns, the authors refer repeatedly to its “activity”. Given that its activity is not being directly assayed, it is more appropriate to refer simply to its protein levels or localization.

6. The discussion of gastrulation and its relationship to Brachyury expression throughout the manuscript is confusing. Because Bra expression precedes the actual ingression of mesoderm in many species, it seems gastrulation onset should be defined as the start of mesoderm ingression rather than Bra expression onset. The authors refer to gastrulation prior to lumenogenesis presumably because Bra is expressed at this time? But it seems clear that gastrulation as defined by mesoderm ingression begins only after lumen formation. In the introduction, the authors further state that lumenogenesis

coincides with an EMT, but no evidence is provided for this beyond Bra expression (which alone does not indicate EMT).

7. The authors also state in the Discussion that AP axial patterning is required for gastrulation, which is not true. Chicken embryos that have been radialized and have no discernible AP axis still produce mesoderm around the circumference of the embryo (Alev et al, Development, 2013) and thus, gastrulate.

8. The authors state that they are the first to report circular expression of Brachyury pre-gastrulation, but Bra (tbxta) is expressed circularly around the embryonic margin of zebrafish embryos, for example, well before gastrulation onset. This statement should be revised to better qualify in what species or conditions such an expression pattern is observed.

9. Without a molecular marker, it is not clear that the trophoblast-like cells identified here are indeed trophoblast. This should be made clear, and these cells should consistently be referred to as “trophoblast-like” rather than “trophoblast”.

10. For each figure, authors should indicate how representative the presented images are (i.e. how many of the embryos examined exhibited the phenotype shown).

Minor comments

1. The image shown in Fig. 2E is quite blurry and the reader cannot easily identify the features that authors point out in the text. The images in panels G and J are much clearer… can a larger image like those be provided instead?

2. When referencing Fig. 5A XIX, the text refers to circle and arrows (site of gastrulation) while the figure and legend refer to asterisks (site of gastrulation) and arrows.

Reviewer #2

(Remarks to the Author)
In this manuscript, Weberling et al present the first morphological characterisation of the pre-oviposition development of the veiled chameleon. This species is not a classical model organism, and very little is known about its early development. The manuscript also provides a useful staging for future studies and will be relevant for those interested in evo-devo and the investigation of non-model organisms. Having said this, the paper would benefit from a molecular characterisation alongside the morphological one, some of the claims need to be toned down, and several clarifications would be useful as follows:

Major comments:
1. The manuscript is focused on the morphology of the cells and tissues of the developing embryos. The authors acknowledge that the lack of reliable markers to label the different tissues is a limitation. This limitation would be overcome by providing a complementary molecular characterisation by for example, single-cell RNA sequencing. There are several instances in the manuscript when the molecular information would be very helpful. The authors mention amniotic cells appearing in the embryos. What is the evidence to support that these are amniotic cells? Similarly, the trophoblast identity of the cells is simply based on size and multinucleation. A molecular characterisation is needed to support these claims. Along similar lines, large and small blastomeres are described at the earlier stages. What is the relevance of this? Are there already different cell types?
2. The authors mention non-adherent blastomeres at stage VI. How do they know the blastomeres are non-adherent? Have they stained for any adhesion proteins? If the cells are forming rosettes, they must somehow adhere to each other.
3. The appearance of an amnion-like structure is not clear. I cannot see squamous cells in Figure 3G. The authors have simply put a blue shadow on top of a specific region of the embryo. A staining of F-actin would help with the characterisation of cell and tissue shapes. Are the authors proposing that there is a squamous epithelium directly on top of the pseudostratified epiblast? I don’t see the amnion in the schematics provided, and it’s difficult to understand the tissue topology from the images provided. Similarly, the gastrulation region in the H&E staining is not very clear. Can’t they provide the same cross-section with a DAPI/laminin/Brachyury staining?
4. In the discussion, the authors claim they have characterised the activity of signalling pathways, but the activity has not been evaluated in this manuscript. They also mention that the putative trophoblast cells are derived from the epiblast, but I do not understand what experimental evidence supports this claim.

Minor comments:
1. The figures would need to be reorganised to follow the developmental timing. For example, Figure 1A is only mentioned in the introduction and not properly discussed. This should be moved to the corresponding developmental stage in the manuscript.
2. I may not have understood the timings of development based on what is explained. The authors mention that all the eggs they isolated between 17-37 dpc were not fertilised. If the average lay date is 37 days dpc, how is it possible that they are studying the pre-oviposition stage?
3. The authors mention that the staging will be useful for future functional studies. It would be interesting to mention in the discussion how those functional studies could be carried out in this species.
4. Figure 2J: It would be good to see the original image without the drawings overlaid.
5. I don’t think the title really reflects the manuscript. It just represents one of the findings.

Reviewer #3

(Remarks to the Author)
This is an interesting study of veiled chameleon early development. The main finding of this work is that lumenogenesis of epiblast is seen in this species, thus the authors concluded that mammalian epiblast luminogenesis (forming epiblast and amnion from an initial epiblast progenitor cell population) in not unique to mammals. The work is largely descriptive. This model has been studied by many others (not all previous work were cited here) and lumenogenesis (or early amniogenesis, equivalent to proamniotic cavity formation in mammals) was previously reported (hence the novelty is limited). This current work, however, does constitute a very thorough study of veiled chameleon early development, and some aspects of their findings are of significance (e.g., early leavage, process of amniogenesis/lumenogenesis; expression of several important genes, especially of Wnt3a and Bra). Overall I think it is an interesting and comprehensive work. I have a few points which they authors may consider addressing in order to improve this work.

1) I think the authors need expression analysis of 1 or 2 pluripotency genes (it is important if the authors want to make a direct comparison with mammalian models.
2) the authors need expression analysis of at least 1 PGC markers (rationale same as above).
3) Fig.1Ei*: nuclear pore may be golgi complex. same issue in the figure legend as well.
4) Fig.S3Ci88: should be Fig.S3Ci**
5) Fig.2G5: are egg cells multinucleated? or are they having delayed cytokinesis (i.e., after final cellularization, did the authors see multinucleated cells at all)?
6) Fig.S5 (stage III, o--> to)
7) Fig.3A, how did the authors conclude when blastomeres become adhesive? Did the authors stain for Ecad expression, for example?
8) Fig.3A: what is indicated by arrowheads?
9) folding process (Fig.3E-G): did the authors have proof that "disorganized" cells are of mesenchymal feature, or just of extremely distorted epithelial organization (as in the case with neural tube formation)?
10) Fig.4B: the autors mentioned two populations of Bra+ cells. Did the authors have section data related to this particular embryo/stage?
11) Fig.4F-I: multinucleated trophoblasts are formed by fusion, not a delayed/failed cytokinesis. Here, it looks more like a delayed cytokinesis. Can the authors clarify?
12) For BMP2 staining, did the authors have section data (at late phase of lumenogenesis)?

Version 1:

Reviewer comments:

Reviewer #1

(Remarks to the Author)
The authors have addressed all of my concerns with these revisions.

Reviewer #2

(Remarks to the Author)
The revised version of the manuscript tones down some of the original claims. The key strength of the manuscript is the comprehensive morphological description of the development of a non-model organism, but no functional claims can be made. As also mentioned in my first revision, I think that the lack of molecular markers is a limitation of this study. While I can understand that a full, complete molecular characterisation across tissues and time is beyond the scope of the present manuscript, additional molecular information would have strengthened the impact of this work.

Reviewer #3

(Remarks to the Author)
The authors made significant modifications to the manuscript. They did not address some of my initial concerns, but overall the manuscript is much improved. I am happy to support the publication of this manuscript in Nature Comm.

Open Access This Peer Review File is licensed under a Creative Commons Attribution 4.0 International License, which permits use, sharing, adaptation, distribution and reproduction in any medium or format, as long as you give appropriate credit to the original author(s) and the source, provide a link to the Creative Commons license, and indicate if changes were made.
In cases where reviewers are anonymous, credit should be given to 'Anonymous Referee' and the source.
The images or other third party material in this Peer Review File are included in the article’s Creative Commons license, unless indicated otherwise in a credit line to the material. If material is not included in the article’s Creative Commons license and your intended use is not permitted by statutory regulation or exceeds the permitted use, you will need to obtain permission directly from the copyright holder.
To view a copy of this license, visit https://creativecommons.org/licenses/by/4.0/

RESPONSES TO REVIEWER COMMENTS

the responses are marked in blue. Manuscript text changes are noted in red.

Reviewer #1 (Remarks to the Author):

Here, Weberling and colleagues examine development of the veiled chameleon before oviposition, including lumen formation in the epiblast and subsequent gastrulation. The study begins with a detailed description of chameleon development, including early embryonic cleavages and cellularization. Through careful analysis of staged embryos, they then describe the formation of tissue folds at the edge of the epiblast that elevate dorsally and then close via a purse-string mechanism to enclose an amniotic cavity with a ventral epiblast and dorsal amnion. Soon after the epiblast lumen closes, the embryo breaks anteroposterior symmetry and mesoderm cells are seen ingressing at the posterior end. This ingression coincides with breakdown of the basement membrane and brachyury expression is observed in the nascent mesoderm, but brachyury expression is first observed prior to ingression. It is initially expressed radially in the future amnion before resolving to the site of gastrulation, exhibiting distinct patterns from other amniotes. The authors characterize mRNA expression of additional signaling molecules, with some exhibiting patterns expected from other species (Nodals) and others show novel expression patterns (Wnt3). This study beautifully describes key developmental stages of an emerging model organism, including staging criteria, providing a valuable resource for the community. It further highlights exciting differences and similarities between reptile, avian, and mammalian embryogenesis that provide key insights into the evolution of development. Some statements were identified that require clarification or additional supportive evidence in this otherwise strong study, which are listed below.

We thank the reviewer for this assessment of our study.

1. In the abstract, the authors state that AP patterning occurs independently of Cerberus and Lefty. While the expression patterns of these genes suggest this is likely true, no functional evidence is provided in support of it. I recommend revising this statement accordingly.

We thank the reviewer for this comment and have revised these statements as follows: "Furthermore, our data suggest that anterior-posterior patterning in veiled chameleons may occur independently of Cerberus and Lefty."

2. The authors repeatedly make claims about tissue tension based on actin staining patterns. I would agree that these patterns are suggestive of increased tension, but not indicative. (Functional tests of tissue recoil after laser ablation, etc would be necessary to make these claims). I recommend revising such statements accordingly.

We thank the reviewer for this comment and agree that only a functional test would provide proof for tissue tension. We attempted laser ablation, but due to the lack of an established *in vitro* culture protocol for pre-oviposition chameleon embryos, we could not successfully perform laser ablation during lumen closure. As such, we decided to focus on actomyosin distribution which is indicative of tension to provide more insight into the lumen closure process. To this end, we stained embryos of consecutive developmental time points during lumen closure for F-actin and phosphorylated myosin light chain 2 (non-muscle myosin), which shows a characteristic increase of actomyosin during lumen closure. We include this novel data in a new Figure 3L (see below) and adjusted the text as follows:

"We next investigated lumenogenesis through whole mount F-actin staining (Figure 3L, S7A). Upon the initiation of hollowing, the central epiblast cells were significantly smaller than outer cells and higher circularity (Figure 3Li, S7Ai,S7B-J) This size difference remained throughout lumenogenesis while the overall circularity decreased. Once the epiblast dome matured, the border cells aligned in concentric rings around the epiblast centre (Figure 3Lii, S7Aii). Then, the border cells elongated and exhibited slightly higher actin levels while phosphorylated myosinII (pMyoII) remained low (3Liii, S7Aiii). Next, supracellular actin rings appeared around the epiblast dome in subsequent stages (Figure S7Aiii-iv, red lines plot profiles). The ring-cells stretched so thin, that individual cells could not be distinguished (Figure S7Aiv,v). Subsequently, these ring-cells disappeared and instead, the epiblast was overlaid and surrounded by an actin ring in direct contact with the large outer cells (Figure 3Lv, arrows). This actin ring appears to be a supracellular actomyosin cable (Figure 3Liv-vi). Actin and pMyoII peaked at the border between epiblast and outer cells on top (Figure 3Liv-vi, plot profiles black lines, S7A, plot profiles red lines). This actomyosin ring constricted further over the epiblast exhibiting even higher actin intensity than the underlying epiblast (Figure 3Lvi, S7Avi). Taken together, we defined another 8 consecutive stages of lumenogenesis stages from the coherent embryo (stage VII) to a the closely annealed, although not-yet closed epiblast lumen (stage XIV) (Figure 3M, S5)."

Furthermore, we added the following sentence in the discussion: "While we hypothesise that the lumen closes via active constriction due to actomyosin localisation, live imaging and functional experiments such as laser ablation are required to provide proof of active constriction."

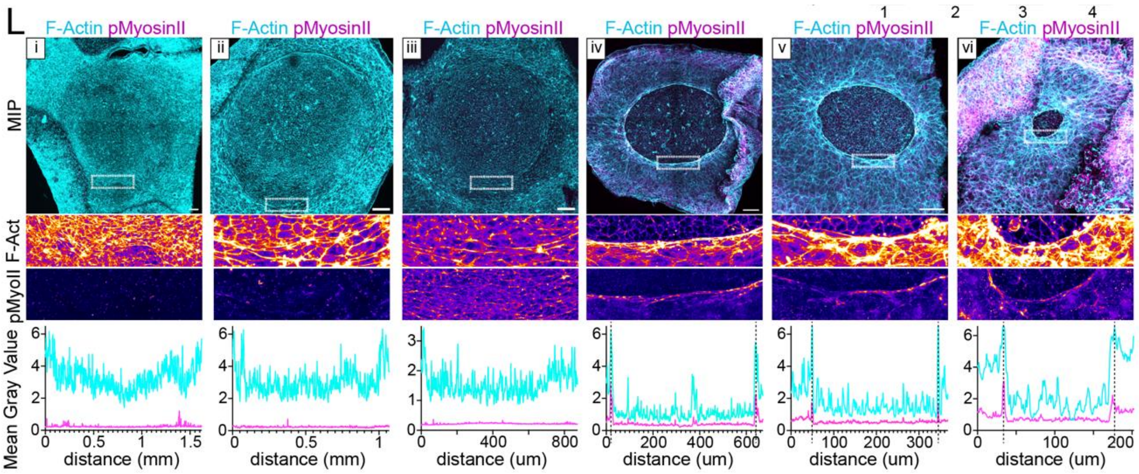

Figure 3L. *top row maximum intensity projections of F-actin (cyan) and phosphorylated Myosin II (pMyosinII, magenta) during lumenogenesis (i-vi) in whole embryos. Dorsal view. squares indicate zoom-in regions of rows 2 (F-actin) and 3 (pMyosinII). Columns iv-vi arrows indicate supracellular actomyosin cable. Bottom row. Plot profiles of actin (cyan) and pMyosinII (magenta) mean gray value drawn across the length of the embryo. black lines indicate the actomyosin cable. Fire staining (yellow-white: high signal intensity, purple-black low signal intensity). Scale bars: 100μm*

3. The authors report the appearance of spindle-shaped ingressing/ ingressed mesoderm cells in the posterior epiblast, but their shape nor their identity is apparent from the EM image shown in Fig. 4B. These cells are much more clearly shown in panels (Fig. 4E) depicting immunofluorescent staining for Brachyury, but their location is not consistent with those outlined in 4B. 4B shows these cells dorsally near the border with the amnion, while they appear more ventrally located in 4E and seem to protrude from the epiblast. Can the authors reconcile these apparently distinct descriptions of the nascent mesoderm?

We thank the reviewer for pointing this out. The embryos do exhibit a natural variation in the precise location of mesoderm ingression upon initiation of gastrulation which may be due to the fact that these are completely outbred chameleons instead of an inbred mouse line. As such, we observed embryos exhibiting mesoderm ingression dorsally directly at the epiblast-amnion border as well as more ventrally. Overall, we did observe a “curling” behaviour of the trophectoderm-like tissue once dissection was completed, which may slightly distort the embryo.
We now include another immunofluorescence panel depicting laminin/brachyury in optical sections (Novel Figure S8A, see below). There, the cell ingression can be observed very close to the epiblast/amnion border. But at the same time, the embryo is overall bent due to the curling of the trophectoderm.

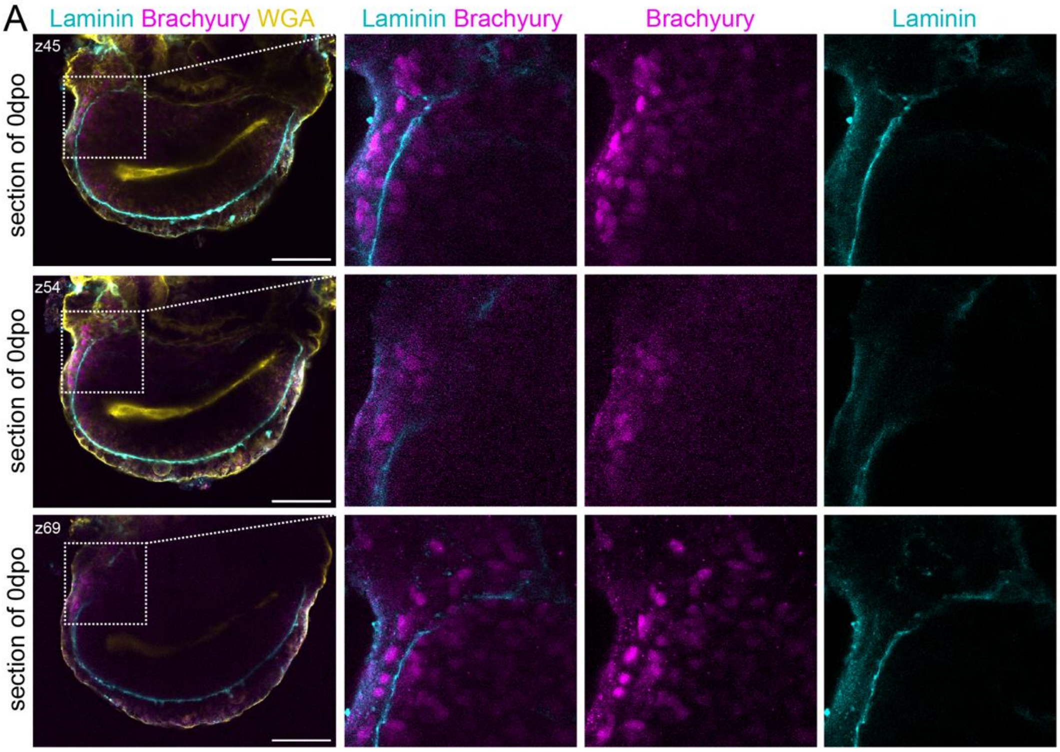

Figure S8: Cell ingression during gastrulation
A. *virtual cross sections of 0dpo gastrulating embryo. row1-3 different cross sections. Laminin (cyan), Brachyury (magenta), WGA (yellow). squares indicate zoom in. All scale bars 100um.*

4. The expression patterns of Brachyury described and shown in fig 5G-H are very interesting, but as presented are somewhat confusing. 5H shows 2 dorsal expression domains at stage XIX, while wholemount staining suggests there should be a single ventral patch at the posterior site of gastrulation. Is the stage of some images labeled incorrectly?

We thank the reviewer for pointing this out. There was indeed a mislabelled panel. Figure 5H shows stage XIII and not XIX. We have corrected this in the Figure.

5. When discussing Brachyury protein expression patterns, the authors refer repeatedly to its “activity”. Given that its activity is not being directly assayed, it is more appropriate to refer simply to its protein levels or localization.

We thank the reviewer for noting this and we have adjusted the text accordingly.

6. The discussion of gastrulation and its relationship to Brachyury expression throughout the manuscript is confusing. Because Bra expression precedes the actual ingression of mesoderm in many species, it seems gastrulation onset should be defined as the start of mesoderm ingression rather than Bra expression onset. The authors refer to gastrulation prior to lumenogenesis presumably because Bra is expressed at this time? But it seems clear that gastrulation as defined by mesoderm ingression begins only after lumen formation. In the introduction, the authors further state that lumenogenesis coincides with an EMT, but no evidence is provided for this beyond Bra expression (which alone does not indicate EMT).

We thank the reviewer for this comment and apologise for the confusion. We agree that Brachyury expression alone does not constitute gastrulation but that mesoderm ingression is required for this distinction. To our knowledge, the only previous description of pre-oviposition development of the veiled chameleon by Karl Peter from 1935 states that gastrulation initiates prior to lumenogenesis based on the apparent delamination of cells during lumenogenesis when the amniotic folds form, while papers focussing on post-oviposition development describe the initiation of gastrulation as just following oviposition. As such we raise that question in the introduction referring to these respective studies. In Figure 4 and S8A, we provide further information on cell shapes, showing the ingression of gastrulating cells at 0dpo. We furthermore tie gastrulation to a break in the basement membrane (Figure 6) thereby showing that while an earlier timepoint for the initiation of gastrulation may have been suggested, we define the initiation of gastrulation around 0dpo, embryonic stage XIX.

7. The authors also state in the Discussion that AP axial patterning is required for gastrulation, which is not true.

Chicken embryos that have been radialized and have no discernible AP axis still produce mesoderm around the circumference of the embryo (Alev et al, Development, 2013) and thus, gastrulate.

We thank the reviewer for this comment. Alev et al. inject FGF4/FGF2/FGF8/ActivinA or Wnt3A into the subgerminal cavity of chicken, quail, emu and japanese soft shell turtle embryos, which results in induction of circular mesoderm. It has also been reported that embryonic stem cells grown in 2D micropatterns can also exhibit radial mesoderm induction following appropriate growth factor simulation. In this case, the inner-most domain becomes the anterior while the posterior is in the outer-most domain resulting in a radial anterior-posterior axis formation instead of a linear one.[1] A similar mechanism may underly the results of Alev et al.. However, to our knowledge amniotes do not gastrulate prior to linear anterior-posterior axis formation *in vivo* without manipulation. In our discussion we do not focus on manipulated embryos. This is why we made this statement.

We adjusted the text as follows to avoid confusion: "While we report Brachyury protein expression at earlier timepoints, we did not observe anterior-posterior patterning which is required for successful gastrulation in amniotes *in vivo* prior to lumenogenesis."

8. The authors state that they are the first to report circular expression of Brachyury pre-gastrulation, but Bra (tbxta) is expressed circularly around the embryonic margin of zebrafish embryos, for example, well before gastrulation onset. This statement should be revised to better qualify in what species or conditions such an expression pattern is observed.

We thank the reviewer for this comment and have revised the text to specify that we are focussing on amniotes as follows: "To our knowledge, we are the first to report pre-gastrulation, circular expression of Brachyury in amniotes."

9. Without a molecular marker, it is not clear that the trophoblast-like cells identified here are indeed trophoblast. This should be made clear, and these cells should consistently be referred to as "trophoblast-like" rather than "trophoblast".

We thank the reviewer for pointing this out, we have already referred to this cell population as trophoblast-like and have now made sure that this is consistent throughout the manuscript.

10. For each figure, authors should indicate how representative the presented images are (i.e. how many of the embryos examined exhibited the phenotype shown).

We thank the reviewer for this comment. We analysed 3-4 embryos per female for each marker or parameter examined. While clutches are roughly time-matched, they are not as time-matched as individual embryos from a mouse litter. As such, it is impossible to obtain precise replicates of each stain other than them coming from the same clutch or multiple equivalent clutches of eggs, especially given the limited total number of embryos we have access to due to the limited number of females typically available in our colony. Wherever we carried out quantification, we provide the exact number of embryos analysed. We included all of this information in the following section in the methods"

"Replicates and Reproducibility
For each experimental parameter, between 3-4 embryos were analysed per female. The embryos of an individual clutch are roughly time-matched but not as identical as found for embryos from a mouse litter. As such, embryos from one female may be distributed across 2-3 developmental stages. For in situ hybridisation experiments, embryos from 8-10 individual females were analysed depending on the probe. For immunofluorescence staining, when focussing on a specific stage, embryos of 1-2 different females were stained. When immunofluorescence staining focused on consecutive stages, embryos of 2-5 females were utilized depending on how many stages were assessed."

Minor comments

1. The image shown in Fig. 2E is quite blurry and the reader cannot easily identify the features that authors point out in the text. The images in panels G and J are much clearer… can a larger image like those be provided instead?

We thank the reviewer for this comment. Figure 2E shows the entire embryonic disk to give the reader an impression of the structure of the whole embryo, while Figure 2G, J are zoom-ins of specific sections of that image to provide more insight into these regions. To better see the details described in the text, we provide zoom-ins of the 5 different areas. While we cannot provide a larger image within the main figures, we now include another embryo in Supplementary Figure S4A as a larger panel (see below).

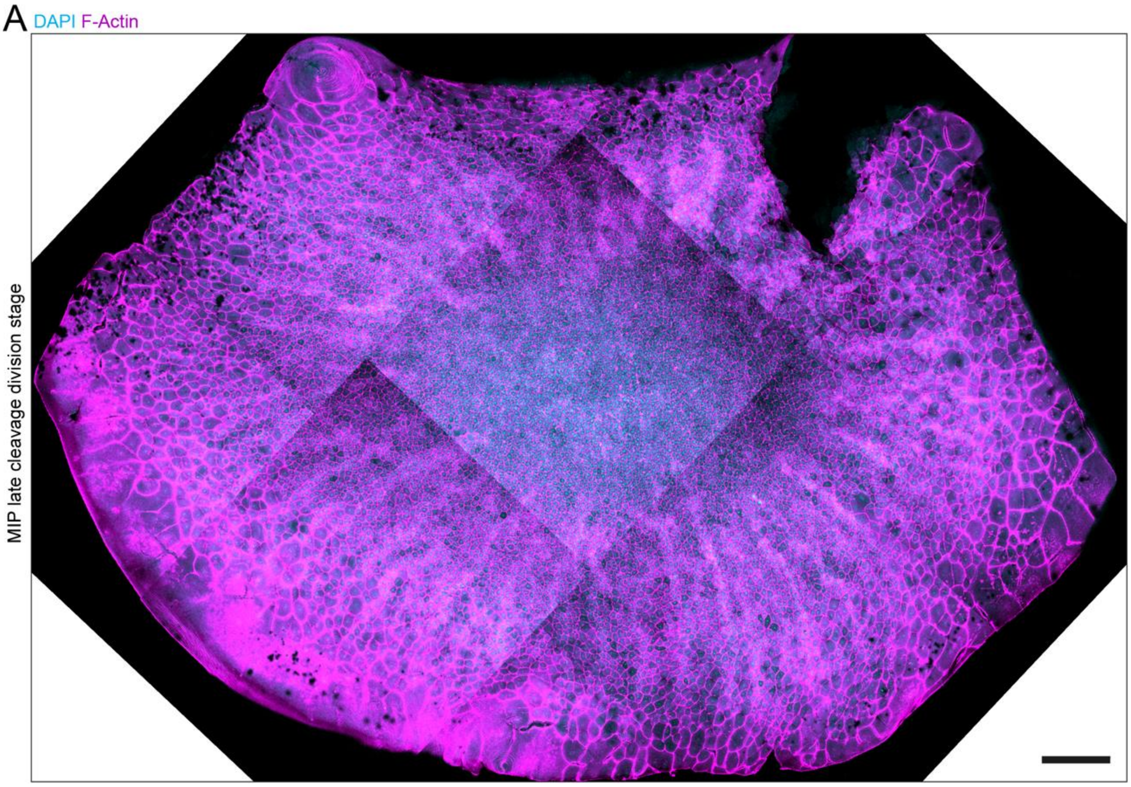

Figure S4A. *maximum intensity projection of late cleavage stage embryo. dorsal view. DAPI (cyan), F-Actin (magenta). replicate to Figure 2E. Scale bar 500um.*

2. When referencing Fig. 5A XIX, the text refers to circle and arrows (site of gastrulation) while the figure and legend refer to asterisks (site of gastrulation) and arrows.

We thank the reviewer for catching this and have changed the text accordingly: "Upon initiation of gastrulation, Cerberus expression divides into 2 domains, one remains in the middle of the embryo (Figure 5A XIX arrows), while the other forms a distinct domain overlying the site of gastrulation (Figure 5A XIX asterisks)."

Reviewer #2 (Remarks to the Author):

In this manuscript, Weberling et al present the first morphological characterisation of the pre-oviposition development of the veiled chameleon. This species is not a classical model organism, and very little is known about its early development. The manuscript also provides a useful staging for future studies and will be relevant for those interested in evo-devo and the investigation of non-model organisms. Having said this, the paper would benefit from a molecular characterisation alongside the morphological one, some of the claims need to be toned down, and several clarifications would be useful as follows:

We thank the reviewer for this assessment of our study. Responses to the individual comments are below.

Major comments:
1. The manuscript is focused on the morphology of the cells and tissues of the developing embryos. The authors acknowledge that the lack of reliable markers to label the different tissues is a limitation. This limitation would be overcome by providing a complementary molecular characterisation by for example, single-cell RNA sequencing. There are several instances in the manuscript when the molecular information would be very helpful. The authors mention amniotic cells appearing in the embryos. What is the evidence to support that these are amniotic cells? Similarly, the trophoblast identity of the cells is simply based on size and multinucleation. A molecular characterisation is needed to support these claims. Along similar lines, large and small blastomeres are described at the earlier stages. What is the relevance of this? Are there already different cell types?

We thank the reviewer for this comment. In this manuscript, we aim to provide a staging system based on morphological analysis of pre-oviposition embryos to lay the basis for molecular and functional studies at specific stages or specific tissues. Carrying out single cell RNA sequencing at multiple timepoints across pre-oviposition development would add sufficient cost and more than a year's worth of additional work to this project and is outside the current scope of this study but rather will be the focus of a follow-up study. While we agree with the reviewer that molecular markers would be helpful when carrying out studies on specific tissues in the future, we

would like to disagree with the claim that tissues cannot be annotated based on their morphology. Mouse and human embryos have been annotated based on their morphology and not based on their tissue marker genes.[2,3] Only few antibodies of highly conserved proteins work on chameleons, as no antibodies to date have been specifically raised against this species. As such, we decided to annotate the tissues found in the chameleon based on their morphology and relative position within the embryo.
The amnion in the chameleon has been described by Peter[4] and discussed as an established fact by Pasteels[5]. We based our annotation on the origin of the tissue, which stems from the epiblast disk (Figure 3F,G), as well as on its position following lumen formation as covering the proximal half of the amniotic cavity while exhibiting characteristic thinning of the tissue (Figure 4A,B).
Regarding the trophoblast, we had annotated it as trophoblast-like in the manuscript as we agree that further studies are required to understand whether this truly is a trophoblast. We have now made sure that this is consistent throughout the text.
Cleavage divisions initiate in the centre of the embryonic disk with the first large blastomeres being formed in the middle of the embryonic disk while the cleavage furrow starts to extend towards the borders of the embryonic disk. While the first large blastomeres at the periphery of the embryonic plate form, the blastomeres in the middle of the embryo continue cleaving, resulting in the size differences between inner and outer blastomeres as noted by the reviewer. This is conserved across avian- and non-avian reptiles[6–8]. Once the cleavage divisions are complete, all blastomeres are of the same size regardless of their relative position within the embryo. As such, blastomere size is a way to understand how far the cleavage divisions have already progressed. Further studies will be required to investigate whether there are differences between inner and outer blastomeres. We would hypothesise for now that once cleavage is completed, the relative position of each blastomere will result in the acquisition of a specific tissue identity similar to inner and outer cells in the mouse morula forming the inner cell mass or the trophoblast. But that needs further investigation.

2. The authors mention non-adherent blastomeres at stage VI. How do they know the blastomeres are non-adherent? Have they stained for any adhesion proteins? If the cells are forming rosettes, they must somehow adhere to each other.

We thank the reviewer for raising these questions. Throughout cleavage stages, it is not possible to dissect the embryo from the underlying yolk without ripping the embryo apart because the individual cells do not adhere to each other. They only form a cohesive sheet from stage VII onwards. As such, up to stage VI the embryos are dissected on top of a yolk plate to ensure tissue integrity. We believe that rosette formation may show that the inner cells initiate stronger adhesion to each other. But we do not have data to support this hypothesis. And a future study will be required to understand the process of adhesion between the individual blastomeres. We have clarified this issue in the method section focussing on dissection:

"Then, incisions were made around the embryonic shield and the shield plus an underlying layer of yolk was isolated using needles from the remaining yolk. This yolk plate is required to maintain the integrity of the embryo as the blastomeres are not cohesive and will otherwise separate."

3. The appearance of an amnion-like structure is not clear. I cannot see squamous cells in Figure 3G. The authors have simply put a blue shadow on top of a specific region of the embryo. A staining of F-actin would help with the characterisation of cell and tissue shapes. Are the authors proposing that there is a squamous epithelium directly on top of the pseudostratified epiblast? I don't see the amnion in the schematics provided, and it's difficult to understand the tissue topology from the images provided. Similarly, the gastrulation region in the H&E staining is not very clear. Can't they provide the same cross-section with a DAPI/laminin/Brachyury staining?

We thank the reviewer for this comment, however Figure 3G highlights the trophoblast and not the amnion. We believe that the amnion only differentiates once the lumen is closed (Figure 4B). So, the focus of Figure 3G is on the squamous tissue that is overlaying the epiblast folds. We added a clean image without highlights in the supplementary information that annotates the squamous tissue overlaying the epiblast folds with arrows in zoom-ins (Figure S6E, below). We also decreased the opacity of our pseudo-colouration to ensure the tissue is better visible.

E

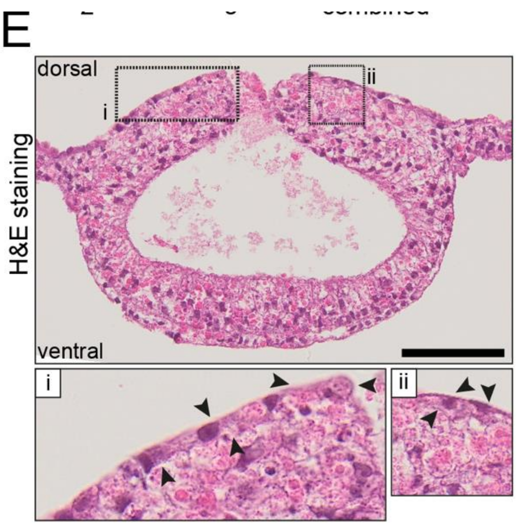

Figure S6E: *H&E staining of embryo during lumenogenesis stages, non-pseudo-coloured image of Figure 3G. squares indicate region of zoom-in. emerging trophoblast-like layer annotated with arrows. scale bar 100µm.*

Overall, we decided to provide histological staining of cross sections and immunofluorescence staining of whole mounts to enable the reader to see the different embryonic stages in as much detail as possible. As such, we show gastrulation in histological sections in Figure 4B and then as whole-mount and virtual sections in Figure 4D/E. We now include a new supplementary panel (Figure S8A) which provides the cross-section view. Please also see our response to Reviewer 1 comment 3.

4. In the discussion, the authors claim they have characterised the activity of signalling pathways, but the activity has not been evaluated in this manuscript. They also mention that the putative trophoblast cells are derived from the epiblast, but I do not understand what experimental evidence supports this claim.

We thank the reviewer for this comment. We have analysed the dynamics and distribution patterns of key signalling pathway components. While we do not present functional proof of pathway activity, dynamic expression of Nodal1/2/Wnt3A in one tissue and the expression of the antagonist Cer1 in another does allow the conclusion that the respective pathways are being activated. To not overstate and thus temper the conclusions that can be drawn from mRNA data alone, we have adjusted our discussion as follows:

"Here, we describe veiled chameleon pre-oviposition morphogenesis from fertilisation to gastrulation and provide evidence of the dynamic spatiotemporal expression of key regulatory genes governing anterior-posterior patterning and gastrulation."

Minor comments:
1. The figures would need to be reorganised to follow the developmental timing. For example, Figure 1A is only mentioned in the introduction and not properly discussed. This should be moved to the corresponding developmental stage in the manuscript.

We thank the reviewer for this point. The data are organised to follow developmental timing, first focussing on morphology (Figures 1-4) to establish a staging system. Then we use this newly established staging system to follow gene expression patterns at the RNA level (Figure 5) and then at a protein level for Laminin and Brachyury (Figure 6) over time. Figure 1A presents the rationale of why we carried out a study on chameleon pre-oviposition development as its morphology diverges from chicken[6,9] or brown anole[8] morphology at a similar developmental stage.

2. I may not have understood the timings of development based on what is explained. The authors mention that all the eggs they isolated between 17-37 dpc were not fertilised. If the average lay date is 37 days dpc, how is it possible that they are studying the pre-oviposition stage?

We thank the reviewer for their comment. We initially hoped that we could stage chameleon embryos based on the days post copulation. However, veiled chameleons store sperm which has been reported for several other squamates but not yet for this species meaning that the day of copulation and the day of fertilisation are uncoupled in contrast to mammalian model systems. 37dpc is an average lay date, but we recorded females laying as early as 13 days post copulation and as late as 251 days post copulation. Since these chameleons are obtained through the pet trade, they are not like inbred mouse lines, which may explain some of the variation. Interestingly, we could not observe this phenomenon in specific individual females. A female that lays very early once may lay much later the next time. As such, counting days post copulation cannot be used to assess embryonic age. This is why we decided to use ultrasound to assess eggshell formation which is only initiated

following fertilisation. This proved to be much more reliable. Eggshell thickness can be correlated with embryonic stage, which allows for collection of embryos at a specific stage-range.

3. The authors mention that the staging will be useful for future functional studies. It would be interesting to mention in the discussion how those functional studies could be carried out in this species.

We thank the reviewer for this input and added the following section:
"Our staging system provides a framework for future functional studies enabling comparability between different investigations, in the same manner as the mouse embryo staging convention of counting embryonic days (E) ensures every reader knows what stage is analysed. This will enable the definition of the effect of *in vitro* culture, exposure to growth factors or inhibitors and potentially gene deletion via CRISPR on morphogenesis."

4. Figure 2J: It would be good to see the original image without the drawings overlaid.

We thank the reviewer for pointing this out and provide now the original image in the supplementary information (Figure S4E) as shown below:

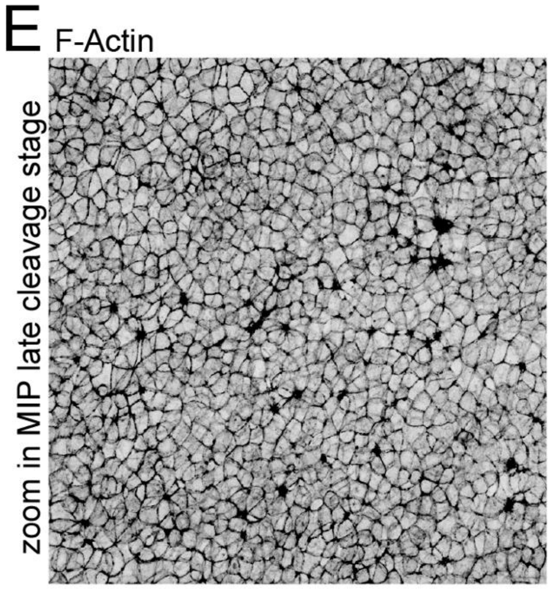

Figure S4E. *Rosettes within the centre of late cleavage stage embryos. Unlabelled image of Figure 2J.*

5. I don't think the title really reflects the manuscript. It just represents one of the findings.

We thank the reviewer for this comment. While we agree that our study has several more findings, we do believe that this is one of the main findings, which is why we chose it for the title to also adhere to the given title length by the journal.

Reviewer #3 (Remarks to the Author):

This is an interesting study of veiled chameleon early development. The main finding of this work is that lumenogenesis of epiblast is seen in this species, thus the authors concluded that mammalian epiblast luminogenesis (forming epiblast and amnion from an initial epiblast progenitor cell population) in not unique to mammals. The work is largely descriptive. This model has been studied by many others (not all previous work were cited here) and lumenogenesis (or early amniogenesis, equivalent to proamniotic cavity formation in mammals) was previously reported (hence the novelty is limited). This current work, however, does constitute a very thorough study of veiled chameleon early development, and some aspects of their findings are of significance (e.g., early cleavage, process of amniogenesis/lumenogenesis; expression of several important genes, especially of Wnt3a and Bra). Overall I think it is an interesting and comprehensive work. I have a few points which they authors may consider addressing in order to improve this work.

We thank the reviewer for this assessment. We aimed to be as complete as possible in citing previous studies of early chameleon embryogenesis. However, to our knowledge, only Karl Peter worked on pre-oviposition veiled chameleons in 1934/35 (in German)[4,10]. We followed his publications up to 1939, but these focussed on other reptiles. The only other publication on pre-oviposition veiled chameleons is by Jean Pasteels in 1957 constituting a review and not a study presenting new data (in French), who mentions the fact that early amniogenesis is very rare in reptiles[5]. Neither of the reptile researchers in the 50s/60s (Hubert, Dufaure, Lemus, Zehr)[11–14] have focussed on veiled chameleons as far as we were able to assess from their publications. All other more recent

studies on veiled chameleons since then focus on post-oviposition stages and development. If the reviewer does have access to another pre-oviposition publication, we would ask them to share the citation.
We cite in our study work focussing on *Mabuya mabouya*, a species that also exhibits epiblast lumen formation.[15] However, we would like to point out that the study cited does not mention the epiblast morphology in any way. It focusses on the placental morphology. We analysed the sections shown and could define an epiblast exhibiting a lumen. We have changed the text in our discussion to reflect this as it may be unclear in our manuscript. Other than that, we are not aware of any study showing epiblast lumen formation prior to gastrulation in a non-avian reptile and would again be very grateful if the reviewer could share any respective citations.

Changed text: "Although not reported in a study focused on placenta formation in *Mabuya mabouya*, a viviparous skink species, it is evident in sections included in that study that epiblast lumenogenesis occurs in *Mabuya mabouya* embryos.[15] Epiblast lumenogenesis therefore has not only evolved in mammals and chameleons. Considering the evolutionary distance between veiled chameleon and *Mabuya mabouya* is 225MYA,[16] leads to us to hypothesise that epiblast lumenogenesis and pre-gastrulation amniogenesis may have evolved multiple times similar to viviparity.[17]"

1) I think the authors need expression analysis of 1 or 2 pluripotency genes (it is important if the authors want to make a direct comparison with mammalian models.

We thank the reviewer for this comment, and we agree that this will be very interesting. However, with this study we aim to provide a morphological framework to enable research focussing on specific stages or tissues. Understanding the pluripotency dynamics in the veiled chameleon will be a novel study which we hope to do in the future.

2) the authors need expression analysis of at least 1 PGC markers (rationale same as above).

We thank and agree with the reviewer. This will also be the focus of a future study that will then be able to compare directly with mouse and chicken embryos to understand whether the origin of PGCs may differ between species. Unfortunately, this is beyond the scope of this study which aims to lay the foundation for such studies.

3) Fig.1Ei*: nuclear pore may be golgi complex. same issue in the figure legend as well.

We thank the reviewer for pointing this out and apologise that it was not clear in the image. The structure the arrows highlight appears to be a nuclear pore complex. There is a Golgi complex to the top right as well. We updated the figure and figure legend to include asterisks for to annotate the Golgi complex (Figure 1Ei*) and provide here an enlarged image for the reviewer.

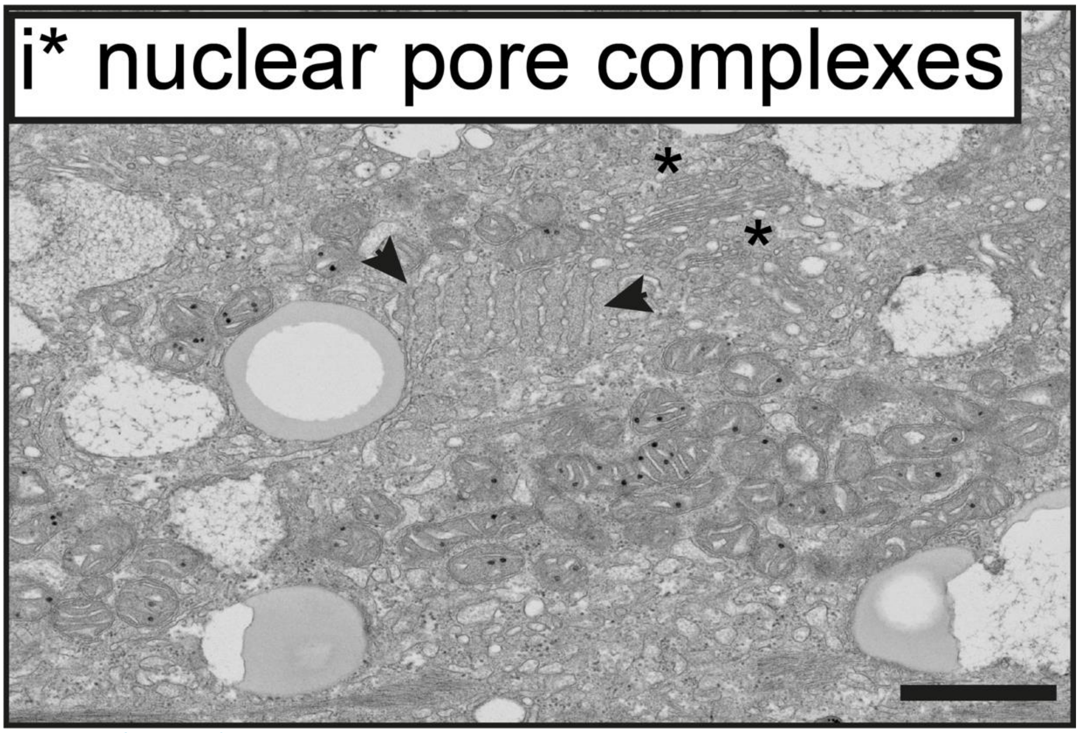

Figure 1Ei*: *TEM of germinal vesicle. (i*) nuclear pore complexes (arrows) and golgi apparatus (asterisks),*

4) Fig.S3Ci88: should be Fig.S3Ci**

We thank the reviewer for spotting that typo and have corrected it.

5) Fig.2G5: are egg cells multinucleated? or are they having delayed cytokinesis (i.e., after final cellularization, did the authors see multinucleated cells at all)?

We thank the reviewer for this very interesting question. Following fertilisation, the embryo undergoes cleavage divisions which coincide with the appearance of cleavage furrows that eventually lead to the cellularisation of the embryo. These furrows appear in some cases later than the nucleus division which results in two nuclei found within one cell boundary during cleavage divisions. This is visible in the border cells of the embryonic disk in Figure 2E,G area 5. Once the cleavage divisions are complete (stage VI), we did not observe any multinucleated cells until the appearance of the trophoblast-like tissue following lumenogenesis (Figure 4F,G).

6) Fig.S5 (stage III, o--> to)

We thank the reviewer for spotting that typo and have corrected it.

7) Fig.3A, how did the authors conclude when blastomeres become adhesive? Did the authors stain for Ecad expression, for example?
We thank the reviewer for this question. The embryo could not be dissected intact during cleavage divisions. Whenever it was attempted to loosen it from the underlying yolk, the cells fell apart. As such, all cleavage divisions were dissected with a thin yolk plate beneath to provide structural integrity of the embryo. Only from stage VII onwards was it possible to isolate the embryo from the yolk. As such, we determined this as the timepoint from which onward the individual embryonic cells adhere to each other. We have adjusted the text in the methodology to clarify that as follows:

"Incisions were made around the embryonic shield and then the shield plus an underlying layer of yolk was isolated from the remaining yolk using needles. This yolk plate is required to maintain the integrity of the embryo as the blastomeres are not cohesive and will otherwise separate."

8) Fig.3A: what is indicated by arrowheads?

The arrowheads are highlighting the tightly packed top cell layer, while the asterisks indicate the loosely packed, spongy cell layer beneath. We propose that the top layer is the nascent epiblast.

9) folding process (Fig.3E-G): did the authors have proof that "disorganized" cells are of mesenchymal feature, or just of extremely distorted epithelial organization (as in the case with neural tube formation)?

We thank the reviewer for this comment. We do not have proof that these cells are of mesenchymal character which is why we only described them as disorganised in the text.

10) Fig.4B: the authors mentioned two populations of Bra+ cells. Did the authors have section data related to this particular embryo/stage?

We thank the reviewer for this comment. The two populations we refer to in Figure 4D are the Bra+ cells that have already ingressed through the basement membrane and those that have not yet ingressed. We now include a new supplementary panel (Figure S8A) that shows a virtual cross-section view. Please also see our response to Reviewer 1 comment 3.

11) Fig.4F-I: multinucleated trophoblasts are formed by fusion, not a delayed/failed cytokinesis. Here, it looks more like a delayed cytokinesis. Can the authors clarify?

We thank the reviewer for this question. To our understanding, multinucleated trophoblasts can form both through fusion as well as through failed/delayed cytokinesis. We have contributed to a study in marmoset that provided live-imaging data to showing that syncytium trophoblast may also form via endoreduplication. A such, we believe that there may be more than one mechanism that can give rise to multinucleated trophoblast cells.[18] However, regarding our data, we have no dynamic information on how these double-nucleated trophoblast-like cells are formed. Following lumenogenesis, all cells of the trophoblast-like tissue are double-nucleated. A future live imaging study will be required to shed light into this very interesting question. But an *in vitro* culture and live imaging system for pre-oviposition chameleon embryos has not yet been established.

We added the following sentence in the discussion to reflect this: "Further studies will be required to understand where this trophoblast-like tissue originates and how binucleation occurs."

12) For BMP2 staining, did the authors have section data (at late phase of lumenogenesis)?

We did try to section our BMP2 stained embryos, unfortunately, we could only get good cross sections of stage XI embryos. These are now included in Figure S9E. Please see below:

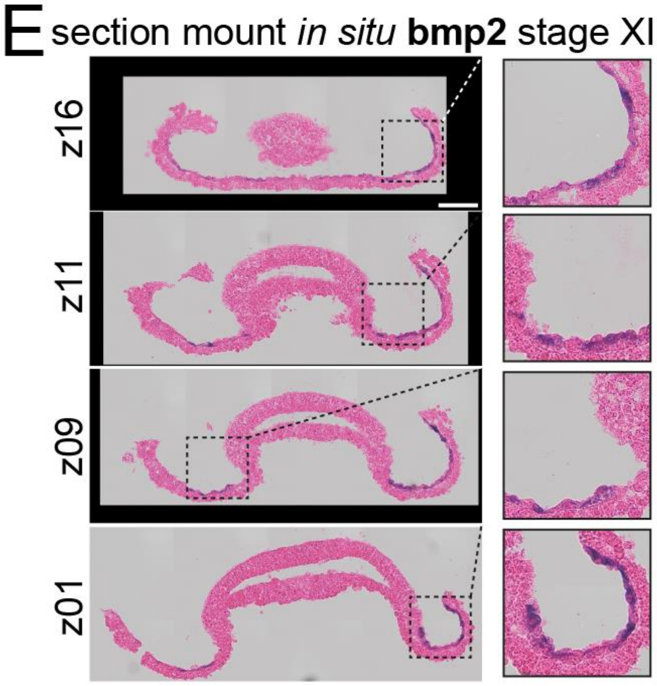

Figure S9E: *cross section of Bmp2 staining at stage XI. Squares indicate regions of zoom-in.*

References:

1. Morgani, S. M., Metzger, J. J., Nichols, J., Siggia, E. D. & Hadjantonakis, A.-K. Micropattern differentiation of mouse pluripotent stem cells recapitulates embryo regionalized cell fate patterning. *eLife* **7**, e32839.
2. Smith, L. J. Embryonic axis orientation in the mouse and its correlation with blastocyst relationships to the uterus: II. Relationships from 4¼ to 9½ days. *Development* **89**, 15–35 (1985).
3. Hertig, A. T., Rock, J. & Adams, E. C. A description of 34 human ova within the first 17 days of development. *Am. J. Anat.* **98**, 435–493 (1956).
4. Peter, K. Die innere Entwicklung des Chamäleonkeimes nach der Furchung bis zum Durchbruch des Urdarms. *Z. Für Anat. Entwicklungsgeschichte* **104**, 1–60 (1935).
5. Pasteels, J. J. La formation de l'amnios chez les Cameleons. *Annales de la Société royale zoologique de Belgique.* **87**, 243–246 (1957).
6. Eyal-Giladi, H. & Kochav, S. From cleavage to primitive streak formation: A complementary normal table and a new look at the first stages of the development of the chick: I. General morphology. *Dev. Biol.* **49**, 321–337 (1976).
7. Sheng, G. Day-1 chick development. *Dev. Dyn.* **243**, 357–367 (2014).
8. Weberling, A. *et al.* Pre-oviposition development of the brown anole (Anolis sagrei). *Dev. Dyn.* **n/a**, 1–23 (2025).
9. Hamburger, V. & Hamilton, H. L. A series of normal stages in the development of the chick embryo. *Dev. Dyn.* **195**, 231–272 (1992).
10. Peter, K. Die erst Entwicklung des Chamäleons (Chamaeleo vulgaris) verglichen mit der der Eidechse (Ei, Keimbildung, Furchung, Entodermbildung). *Zeitschr. f. d. ges. Anat.* **103**, 147–188 (1934).
11. Dufaure, J. P. & Hubert, J. Table de developpement du lezard vivipare: Lacerta Zootoca vivipara jacquin. *Arch Anat Microsc Morphol Exp* 309–328 (1961).
12. Hubert, J. & Dufaure, J. Table de developpement de la vipere aspic: Vipera aspis L. *Bull. Soc. Zool. Fr.* https://www.semanticscholar.org/paper/Table-de-developpement-de-la-vipere-aspic%3A-Vipera-L-Hubert-Dufaure/0b678cc7c6773fe026328b35e0b8b230800e4137 (1968).
13. Zehr, D. R. Stages in the Normal Development of the Common Garter Snake, Thamnophis sirtalis sirtalis. *Copeia* **1962**, 322–329 (1962).
14. Lemus, D. Contribución al estudio de la embriología de reptiles chilenos. II. Tabla de desarrollo de la lagartija vivípara Liolaemus gravenhorti (Reptilia-Squamata-Iguanidae). *Biológica* 39–61 (1967).
15. Jerez, A. & Ramírez-Pinilla, M. P. Morphogenesis of extraembryonic membranes and placentation in Mabuya mabouya (Squamata, Scincidae). *J. Morphol.* **258**, 158–178 (2003).
16. Vidal, N. & Hedges, S. B. The phylogeny of squamate reptiles (lizards, snakes, and amphisbaenians) inferred from nine nuclear protein-coding genes. *C. R. Biol.* **328**, 1000–1008 (2005).

17. Warren, W. C. & Grutzner, F. The Evolution of Viviparity in Vertebrates. in *Placentation in Mammals: Tribute to E.C. Amoroso's Lifetime Contributions to Viviparity* (eds Geisert, R. D. & Spencer, T.) 7–19 (Springer International Publishing, Cham, 2021). doi:10.1007/978-3-030-77360-1_2.

18. Siriwardena, D. *et al.* Marmoset and human trophoblast stem cells differ in signaling requirements and recapitulate divergent modes of trophoblast invasion. *Cell Stem Cell* **31**, 1427-1446.e8 (2024).

REPONSES TO REVIEWERS

Responses to reviewers are marked in blue. Manuscript changes are annotated in red.

Reviewer #1 (Remarks to the Author):

The authors have addressed all of my concerns with these revisions.

We thank the reviewer for this assessment.

Reviewer #2 (Remarks to the Author):

The revised version of the manuscript tones down some of the original claims. The key strength of the manuscript is the comprehensive morphological description of the development of a non-model organism, but no functional claims can be made. As also mentioned in my first revision, I think that the lack of molecular markers is a limitation of this study. While I can understand that a full, complete molecular characterisation across tissues and time is beyond the scope of the present manuscript, additional molecular information would have strengthened the impact of this work.

We thank the reviewer for their assessment. In our study, we describe the morphology of consecutive stages, gene expression patterns of key regulatory genes and hypothesise possible mechanisms. We agree that the next step will be to identify tissue-specific marker genes, perform in-depth transcriptomics analysis over time and eventually establish gene editing to unravel the mechanisms mediating key developmental steps. However, until gene editing of chameleons, and *in vitro* culture of chameleon embryos has been established, such studies cannot be performed. We are therefore already currently working on each of these next vital steps to be able to gain a better functional understanding of chameleon development.
However, such future studies require a precise, morphological framework similar to the embryonic staging systems of other species such as the embryonic day[1]/Theiler[2] stages for mouse, and the Eyal-Giladi-Kochav[3] and Hamburger-Hamilton[4] stages for chicken. This is one of the main results we present here.

A dedicated section in the method chapter addresses these points:

Limitations of working with a non-model organism
Many tools that are standard for model organisms such as mouse, chicken or frog have not been established in chameleons. For example, no commercially available chameleon-specific antibodies exist. We tested a number of tissue marker antibodies, such as Oct4, Sox2, Cdx2, Gata6, and Gata4 that showed either no signal or expression patterns that diverge from what has been published for the species they have been raised against. Only for highly conserved genes such as Laminin and β-catenin could we detect signals that appear specific and were in accordance with protein localisation patterns in other species. Furthermore, we did perform gene expression analyses where we could for Nodal1, Nodal2, Cerberus, Lefty, Brachyury, Wnt3A, and Bmp2. In addition, common tools to enable functional experiments such as trangenesis or chemical agonist/antagonist treatment of in vitro cultured pre-oviposition embryos have not yet been established. As such, we cannot currently provide mechanistic proof for hypothesised cell and tissue movements.

We have furthermore clarified in relevant sections in the discussion that this study provides the foundation or basis for future functional studies.

"Our staging system provides a framework for future functional studies enabling comparative analyses throughout development, in the same manner as the mouse embryo staging convention of counting embryonic days (E) ensures every reader knows what stage is analysed. This will enable the effects of in vitro culture, exposure to growth factors or inhibitors and potentially gene deletion via CRISPR/Cas gene editing or overexpression transgenesis on morphogenesis to be determined."

"… future studies of cell behaviour and transcriptomics will be required. Additionally, a comparison of signalling dynamics between species with and without epiblast lumenogenesis is needed to shed light on the role of the lumen in subsequent development."

Reviewer #3 (Remarks to the Author):

The authors made significant modifications to the manuscript. They did not address some of my initial concerns, but overall the manuscript is much improved. I am happy to support the publication of this manuscript in Nature Comm.

We thank the reviewer for this assessment.

References:

1. Kaufmann, M. H. *The Atlas of Mouse Development*. (1992).
2. Theiler, K. *The House Mouse; Development and Normal Stages from Fertilization to 4 Weeks of Age.* (Springer Verlag, 1972).
3. Eyal-Giladi, H. & Kochav, S. From cleavage to primitive streak formation: A complementary normal table and a new look at the first stages of the development of the chick: I. General morphology. *Dev. Biol.* **49**, 321–337 (1976).
4. Hamburger, V. & Hamilton, H. L. A series of normal stages in the development of the chick embryo. *Dev. Dyn.* **195**, 231–272 (1992).
